# Boosting NAD^+^ blunts TLR4-induced type I IFN in control and systemic lupus erythematosus monocytes

**DOI:** 10.1172/JCI139828

**Published:** 2022-03-01

**Authors:** Jing Wu, Komudi Singh, Amy Lin, Allison M. Meadows, Kaiyuan Wu, Vivian Shing, Maximilian Bley, Shahin Hassanzadeh, Rebecca D. Huffstutler, Mark S. Schmidt, Luz P. Blanco, Rong Tian, Charles Brenner, Mehdi Pirooznia, Mariana J. Kaplan, Michael N. Sack

**Affiliations:** 1 Laboratory of Mitochondrial Biology and Metabolism,; 2 Bioinformatics and Computational Core Facility, and; 3 Cardiovascular Branch, National Heart, Lung, and Blood Institute (NHLBI), NIH, Bethesda, Maryland, USA.; 4 Department of Biochemistry, Carver College of Medicine, University of Iowa, Iowa City, Iowa, USA.; 5 Systemic Autoimmunity Branch, National Institute of Arthritis and Musculoskeletal and Skin Diseases (NIAMS), NIH, Bethesda, Maryland, USA.; 6 Mitochondria and Metabolism Center, Department of Anesthesiology and Pain Medicine, University of Washington School of Medicine, Seattle, Washington, USA.; 7 Department of Diabetes and Cancer Metabolism, Beckman Research Institute, City of Hope, Duarte, California, USA.

**Keywords:** Inflammation, Metabolism, Autophagy, Cytokines, Innate immunity

## Abstract

**BACKGROUND:**

Fasting and NAD^+^-boosting compounds, including NAD^+^ precursor nicotinamide riboside (NR), confer antiinflammatory effects. However, the underlying mechanisms and therapeutic potential are incompletely defined.

**METHODS:**

We explored the underlying biology in myeloid cells from healthy volunteers following in vivo placebo or NR administration and subsequently tested the findings in vitro in monocytes extracted from patients with systemic lupus erythematosus (SLE).

**RESULTS:**

RNA-Seq of unstimulated and LPS-activated monocytes implicated NR in the regulation of autophagy and type I IFN signaling. In primary monocytes, NR blunted LPS-induced IFN-β production, and genetic or pharmacological disruption of autophagy phenocopied this effect. Given that NAD^+^ is a coenzyme in oxidoreductive reactions, metabolomics was performed and identified that NR increased the inosine level. Inosine supplementation similarly blunted autophagy and IFN-β release. Finally, because SLE exhibits type I IFN dysregulation, we assessed the NR effect on monocytes from patients with SLE and found that NR reduced autophagy and IFN-β release.

**CONCLUSION:**

We conclude that NR, in an NAD^+^-dependent manner and in part via inosine signaling, mediated suppression of autophagy and attenuated type I IFN in myeloid cells, and we identified NR as a potential adjunct for SLE management.

**TRIAL REGISTRATION:**

ClinicalTrials.gov registration numbers NCT02812238, NCT00001846, and NCT00001372.

**FUNDING:**

This work was supported by the NHLBI and NIAMS Intramural Research divisions.

## Introduction

Caloric restriction, intermittent fasting, time-restricted feeding, and fasting mimetic diets confer immunomodulatory effects, including amelioration of inflammatory diseases (1–6) and blunting of the NLRP3 inflammasome, circulating cytokines, and acute-phase reactant levels (7–9). In contrast, obesity predisposes to enhanced inflammation (10, 11). Diet-linked mediators of nutrient load–dependent immunomodulation include fasting-induced ketogenesis (1, 12), feeding and obesity-associated endotoxemia (13, 14), and a role for acute dietary changes on the gut microbiome in modulating levels of fatty and bile acid mediators of inflammation (15). Additionally, caloric restriction promotes homing of leukocytes to the bone marrow (16). Despite these observations, the direct roles of caloric load on circulating immune cell homeostasis and activation are not well explored (17).

To characterize the effects of restrictive dietary interventions, we employed an acute model of 24-hour fasting compared with 3 hours of refeeding in healthy volunteers to explore effects on circulating immune cell function. Fasting blunted the NLRP3 inflammasome, and refeeding independently primed and activated this sterile inflammatory program (18). In a subsequent study in steroid-naive individuals with asthma, these findings were validated and additionally showed that fasting and refeeding had modulatory effects on CD4^+^ effector T cells and on respiratory epithelial cell inflammation (19). Furthermore, the fasting effect on CD4^+^ T cells was mediated, in part, via transcriptional regulatory control (20). In the initial healthy volunteer study, the NAD^+^ precursor nicotinamide riboside (NR; ref. 21) similarly blunted the NLRP3 inflammasome in isolated human PBMCs and macrophages (18). Furthermore, nicotinamide mononucleotide (NMN), a biosynthetic intermediate in the same NAD^+^ salvage pathway, suppressed gene signatures of age-associated adipose tissue inflammation in mice (22), and NR reduced neuroinflammation in a murine model of Alzheimer’s disease (23), depressed inflammatory cytokines in older men in a placebo-controlled trial (24), and reduced inflammation in circulating PBMCs in patients with heart failure (25). Moreover, coronavirus infection was recently shown to induce an IFN response that depressed cellular NAD^+^ in a manner that was opposed by supporting NAD^+^ status (26). These findings and the potential use of NAD^+^ booster NR as a therapeutic supplement support further study into the regulatory mechanisms underpinning the NR effect on immune cells.

Activated innate immune cells, in particular circulating monocytes and tissue macrophages, are first responders in pathogen clearance through a range of actions that ultimately resolve inflammation and support tissue homeostasis. Given these emerging findings, we proposed that employing unbiased strategies to explore the effect of NR on monocytes would be a useful approach to begin to understand the broader roles of NAD^+^ supplementation on circulating innate immune cell function. Because refeeding is proinflammatory (18, 19) and overfeeding disrupts hepatic NAD^+^ metabolism (27), we focused this study on exploring the antiinflammatory effects of NR in monocytes extracted from study participants in the refed state.

Here, we (a) performed a double-blinded, placebo-controlled study to explore the effect of NR supplementation in healthy volunteers; (b) generated RNA-Seq libraries to study transcript changes in monocytes extracted from volunteers administered the placebo or NR; (c) employed bioinformatic pathway enrichment analysis, which uncovered that NR modulated autophagy in naive and LPS-activated monocytes and blunted LPS-induced type I IFN signaling in activated monocytes; (d) used biochemical, pharmacological, and genetic manipulation studies to functionally confirm the role of NAD^+^ in regulating autophagy and type I IFN effects; (e) employed metabolomic analysis to identify that NR increased levels of pentose-phosphate pathway (PPP) and purine biosynthetic intermediates; (f) functionally validated that the purine intermediate, inosine, blunted autophagy and type I IFN secretion; and (g) identified that these effects of NR were operational in modulating autophagy and the type I IFN pathway in monocytes from individuals with systemic lupus erythematosus (SLE).

## Results

### NR supplementation modifies gene expression patterns in naive and activated monocytes.

**Figure 1:**
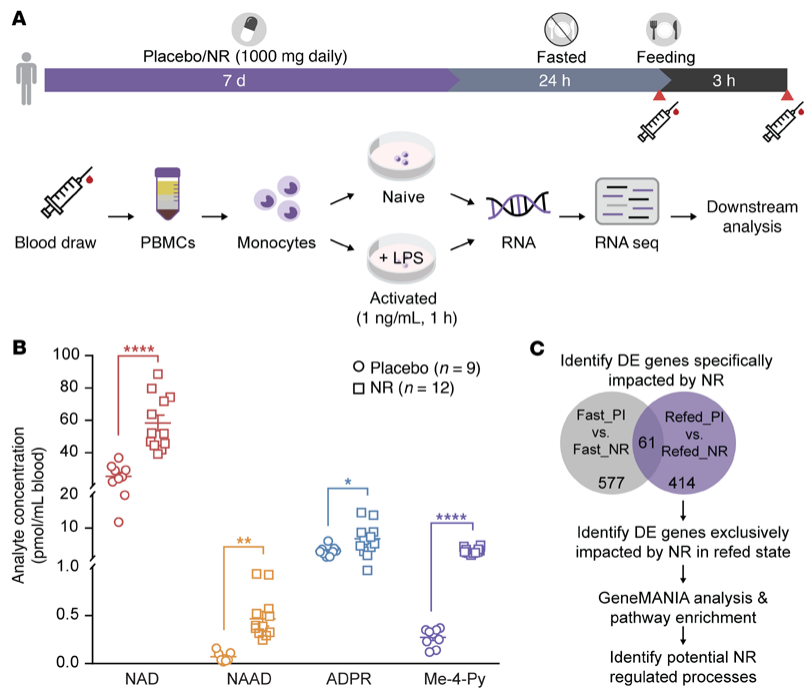
Study design and NAD^+^ metabolome in study participants. (**A**) The clinical protocol depicted in the horizontal bar shows volunteers consuming NR or placebo for 7 days followed by 24-hour fasting and 3 hours of refeeding. The syringe symbol depicts blood draw time points for monocyte isolation and RNA-Seq. (**B**) Elevation of the whole-blood NAD^+^ metabolome following oral NR in participants measured by liquid chromatography-mass spectrometry (LC-MS) (*n =* 9 placebo and *n =* 12 NR). Data analyzed by unpaired 2-tailed Student’s *t* test. *P < 0.05; **P < 0.01; and ****P < 0.0001. (**C**) Flowchart showing fasting and refeeding placebo versus NR data sets used to identify NR-modulated genes specifically affected during refeeding. The 414 NR-exclusive DE genes were subjected to pathway enrichment and geneMANIA analysis. NAD, nicotinamide adenine dinucleotide; NAAD, nicotinic acid adenine dinucleotide; ADPR, ADP ribosyl metabolite; Me-4-Py, N-methyl-4-pyridone-5-carboxamide.

Monocytes isolated from individuals consuming the placebo or NR were used to extract RNA and to prepare RNA-Seq libraries (Figure 1A and Supplemental Figure 1A). The basal characteristics and complete blood cell counts of the healthy volunteers are shown in Supplemental Tables 1 and 2 (supplemental material available online with this article; https://doi.org/10.1172/JCI139828DS1). Fasting insulin levels were less than 50% of the insulin level 3 hours after the fixed caloric meal in this young, healthy cohort, supporting the integrity of the fast (Supplemental Figure 1B). The levels of NAD^+^ metabolites (28) were uniformly elevated in whole blood from the NR versus placebo group, confirming study compliance (Figure 1B). The pipeline used to analyze the FASTA files from RNA-Seq is illustrated in Supplemental Figure 1C. The study design included isolation of monocytes from the NR and placebo group participants at the end of the 24-hour fast and 3 hours after refeeding to enable analysis of the effect of NR in the fasted and refed states (Figure 1A). The isolated naive monocytes and LPS-stimulated monocytes (activated) from both caloric-load states were employed for RNA-Seq analysis. These RNA-Seq databases are accessible in NCBI’s Gene Expression Omnibus (GEO GSE192536). Differentially expressed (DE) genes showed NR-responsive genes in naive and activated monocytes under both caloric-load conditions (Supplemental Figure 2, A and D). Similarly, DE genes from refed versus fasted comparisons showed nutrient load–dependent gene expression change (Supplemental Figure 2, B, C, E, and F). Furthermore, principal component analysis (PCA) plots of the DE genes showed that NR supplementation and nutrient load were the principal components driving gene expression changes, irrespective of monocyte activation status (Supplemental Figure 2, G–L).

To dissociate the overlap of fasting and NR effects on NAD^+^, we exclusively focused on the role of NR on immunometabolism by investigating naive and activated monocytes extracted from the refed groups. Interestingly, pathway enrichment analysis of the DE genes from the naive monocyte comparison between the placebo and NR groups revealed significant enrichment in lysosome-, vacuole-, or secretory granule–related processes and autophagy (Supplemental Figure 2M). Concomitantly, the DE genes in LPS-activated monocyte comparisons revealed enrichment in several IFN signaling pathways, including IFN-γ and type I IFN signaling, and in transcripts encoding autophagy mediators (Supplemental Figure 2N).

**Figure 2:**
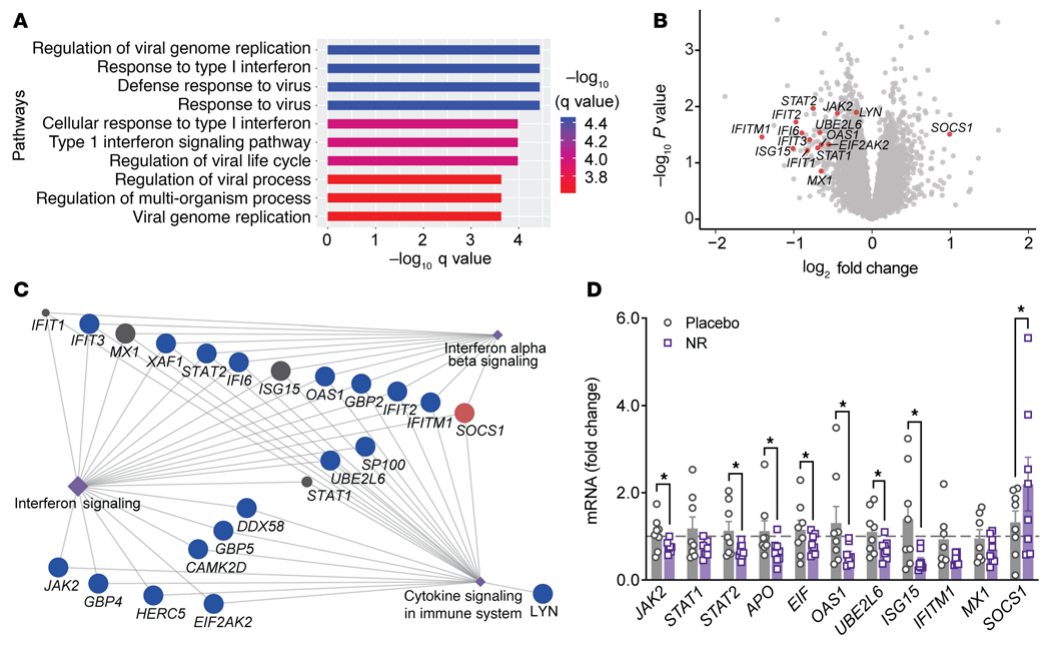
Bioinformatic characterization of RNA-Seq data. (**A**) Pathway enrichment results showing top 10 DE gene pathways modulated by NR in response to LPS stimulation. The *x* axis represents negative log_10_-transformed *q* values where bar plot color is scaled to transformed *q* values. (**B**) Volcano plot showing all the genes from the refed placebo versus NR comparison after LPS activation. The statistically significant type I IFN genes are highlighted in red circles and labeled. (**C**) GeneMANIA consolidated pathway network analysis shows IFN pathway genes (circular nodes) connected to the indicated pathway hubs (diamond-shaped node). The fold-change information is overlaid on this network. NR-linked downregulated genes are represented as blue nodes and the upregulated genes as red nodes. (**D**) Quantitative RT-PCR analysis of type 1 IFN pathway–related genes in monocytes from healthy volunteers taking a placebo or NR for 7 days (*n =* 7–8 individuals/group). Data were normalized to *18S* rRNA and are represented as mean ± SEM. *P < 0.05. Unpaired 2-tailed Student’s *t* test.

To specifically uncover NR-regulated genes in refeeding, DE genes from comparison of the NR and placebo groups after refeeding were overlaid with DE genes from the same comparison under fasted conditions in activated monocytes. This approach is schematized in Figure 1C. The pathway enrichment analysis of this filtered DE gene list revealed significant enrichment of type I IFN signaling (Figure 2A). Overlaying the fold-change information after GeneMANIA analysis revealed that most of these type I IFN–linked transcripts were downregulated by NR with the exception of SOCS 1, a known negative regulator of type I IFN signaling, that was upregulated (Figure 2B). Subsequent bioinformatic analysis supported the hypothesis that NR reduced type I IFN signaling in activated monocytes (Figure 2C), and quantitative PCR (qPCR) validation studies supported that NR diminished transcript levels of the type I IFN–simulated genes in activated monocytes (Figure 2D).

### NR blunts monocytic type 1 IFN signaling and IFN-β production.

**Figure 3:**
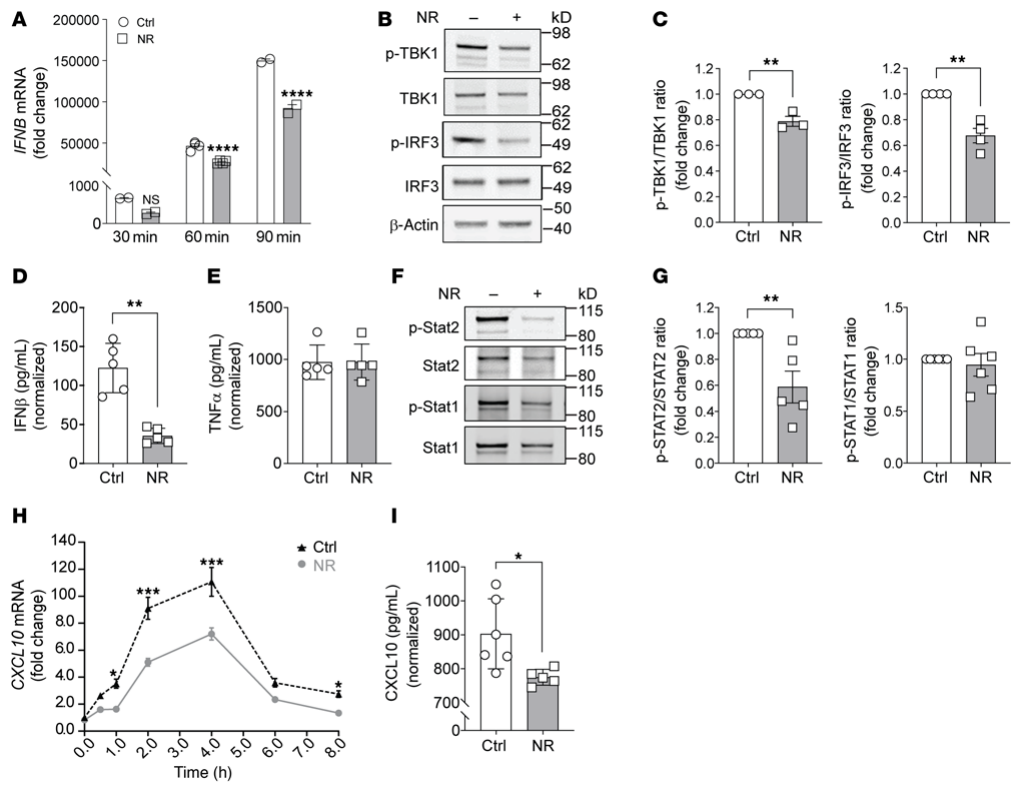
NR administration to human monocytes suppresses LPS-mediated type 1 IFN signaling. (**A**) *IFNB* mRNA level in monocytes was measured by RT-PCR at 30, 60, and 90 minutes after LPS stimulation (*n =* 2–4 replicates). (**B**) Representative immunoblot of phospho-TBK1 and phospho-IRF3 levels in human monocytes preincubated overnight with vehicle or NR (0.5 mM) followed by LPS stimulation (10 ng/mL for 90 minutes). (**C**) Quantification of phospho-TBK1 or phospho-IRF3 level in vehicle- or NR-incubated monocytes normalized to total TBK1 or IRF3 levels (*n =* 3–4 replicates from 3 experiments). (**D**) LPS-stimulated (10 ng/mL for 8 hours) IFN-β production measured by ELISA from human monocytes incubated with vehicle or NR (0.5 mM) for 24 hours (*n =* 5 replicates/treatment; representative results from 3 experiments). (**E**) TNF-α production measured by ELISA from human monocytes incubated with vehicle or NR for 16 hours (*n =* 5 replicates/treatment). (**F**) Representative immunoblot of phospho-STAT2 and phospho-STAT1 levels in human monocytes preincubated overnight with vehicle or NR followed by LPS stimulation. (**G**) Quantification of phospho-STAT2 or phospho-STAT1 level in vehicle- or NR-incubated monocytes normalized to total STAT2 or STAT1 level (*n =* 5–6 replicates from 3 experiments). (**H**) *CXCL10* mRNA level was measured by RT-PCR at different time points upon LPS stimulation. (**I**) CXCL10 production was measured by ELISA from human monocytes incubated with vehicle or NR for 24 hours (*n =* 6 replicates/treatment; representative results from 3 experiments). Data were analyzed by unpaired 2-tailed Student’s *t* test. All data are represented as mean ± SEM. *P < 0.05; **P < 0.01; ***P < 0.001; ****P < 0.0001.

To determine whether NR modulates type I IFN signaling, monocytes isolated from healthy volunteers were cultured in the presence or absence of NR in response to LPS (29). Firstly, the temporal effect of acute LPS administration on the transcript levels of IFN-β was assessed. NR blunted IFN-β expression at 30, 60, and 90 minutes of LPS exposure (Figure 3A). Next, to examine whether NR regulated IFN-β pathway activation, Western blotting was performed to assess phosphorylation and activation of the LPS-activated kinase tank-binding kinase 1 (TBK1) and the downstream transcription factor IRF3 (IFN regulatory factor 3; ref. 6), which then directly transactivates the IFN-β promoter locus. After 90 minutes of LPS, there were no changes in the steady-state levels of TBK1, although its phosphorylation/activation was attenuated by NR (Figure 3, B and C). In parallel, the steady-state levels of IRF3 were also unchanged, whereas NR reduced the phosphorylation of IRF3 (Figure 3, B and C). To evaluate whether NR modulated secretion of IFN-β, the levels of this cytokine were assayed 8 hours after monocytic exposure to LPS. In parallel with the NR effects on type I IFN signaling, IFN-β levels were significantly reduced by NR (Figure 3D). In contrast, in response to LPS, the levels of TNF-α secretion were similar irrespective of NR exposure (Figure 3E). Furthermore, IFN-β stimulation led to phosphorylation and activation of STAT1 and/or STAT2 that control gene expression of a different set of IFN stimulatory genes (ISGs). In monocytes exposed to NR, the extent of STAT2 phosphorylation was significantly attenuated (Figure 3, F and G). To further validate the effect of NR on this pathway, we assayed the transcript levels and secretion of a type I IFN–responsive chemokine, *CXCL10*. Here too, NR reduced *CXCL10* transcript levels (Figure 3H) and chemokine secretion (Figure 3I).

### Disruption of autophagy phenocopies the ameliorative effect of NR on type 1 IFN.

**Figure 4:**
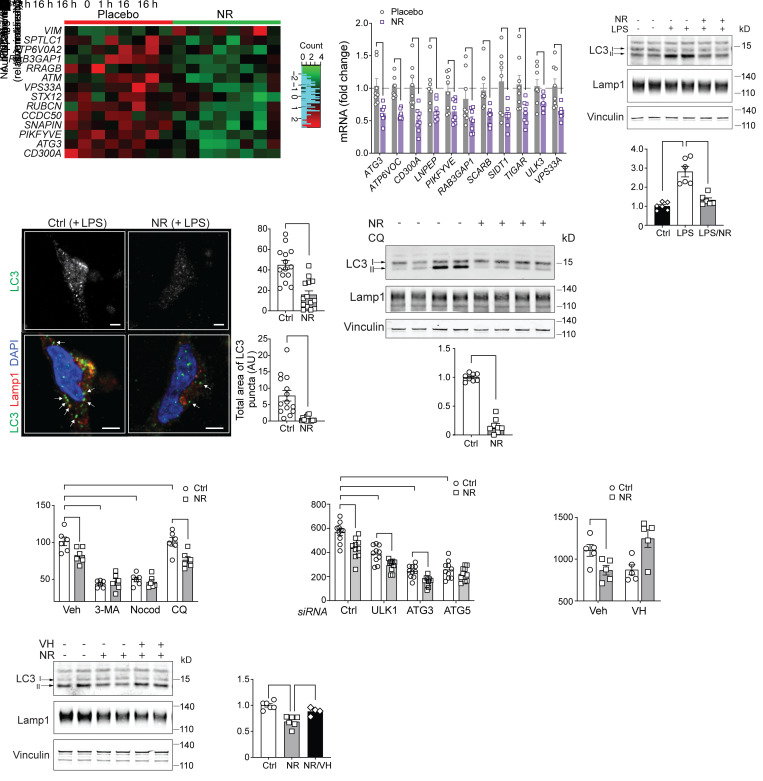
NR-regulated autophagy controls monocytic IFN-β production. (**A**) Autophagy DE gene heatmap comparing LPS-activated placebo and NR-supplemented groups. (**B**) qRT-PCR analysis of monocytic autophagy-related genes from placebo and NR-supplemented groups (*n =* 8 individuals/group). (**C**) Representative immunoblot and quantification of LC3 (I and II) in monocytes preincubated overnight with vehicle or NR followed by LPS stimulation (10 ng/mL for 2 hours). (*n =* 6, 3 experiments). (**D**) Representative confocal images showing LC3 puncta (green) and Lamp1 puncta (red) in monocytes preincubated overnight with vehicle or NR followed by LPS stimulation. Arrows indicate cytoplasmic LC3 puncta. Scale bar: 2 μM. Quantification of total number and area of LC3 puncta in vehicle- or NR-incubated monocytes (*n =* 14 cells/group). (**E**) Representative immunoblot of LC3-II accumulation in response to chloroquine comparing vehicle or NR-supplemented monocytes in response to LPS and quantification of autophagic flux (*n =* 8, 4 experiments). (**F**) LPS-stimulated (10 ng/mL for 6 hours) IFN-β production from monocytes preincubated with autophagosome inhibitors (3-MA or nocodazole) or lysosome inhibitor chloroquine (CQ) in combination with vehicle or NR for 16 hours (representative results from 3 experiments). (**G**) IFN-β production measured by ELISA from monocytes transfected with control siRNA or siRNAs against multiple autophagy genes (representative results from 3 experiments). (**H**) IFN-β production in monocytes preincubated overnight with vehicle or NR or NR + VH (autophagy activator) followed by LPS stimulation (representative results from 3 experiments). (**I**) Representative immunoblot and quantification of LC3-II in human monocytes pretreated with vehicle or NR or NR + VH overnight followed by LPS stimulation. (*n =* 4–6, 3 experiments). Data analyzed using unpaired 2-tailed Student’s *t* test (**B**, **D** and **E**) or 1-way ANOVA followed by Dunnett’s multiple-comparison test (**C** and **I**) or 2-way ANOVA followed by Tukey’s multiple-comparison test (**F** to **H**). All data represented as mean ± SEM. *P < 0.05; **P < 0.01; ***P < 0.001; ****P < 0.0001.

In addition to modulating type I IFN signaling, NR also affected autophagy and autophagy-utilizing processes in naive and LPS-activated monocytes (Supplemental Tables 3 and 4 and Supplemental Figure 2M). A heatmap of curated genes encoding autophagy pathway factors from activated monocytes extracted from the placebo and NR-supplemented groups showed that NR had a profound effect on reducing these transcript levels, with the exception of vimentin (*VIM*), which is known to be a negative regulator of autophagy through the sequestration of beclin-1 (ref. 30 and Figure 4A). qPCR analysis of the study participants’ mRNA validated that the transcript levels of numerous autophagy-linked genes were blunted by NR supplementation in vivo (Figure 4B). Furthermore, the incubation of primary monocytes with NR blunted lipidation of LC3-II (Figure 4C), where the cytoskeletal protein vinculin was employed as a loading control. In parallel, LPS-induced LC3 punctae as measured by super-resolution immunofluorescent microscopy were similarly blunted by NR (Figure 4D). This reduction of autophagosome assembly by NR was confirmed by the temporal measurement of LC3-II accumulation in response to chloroquine administration comparing vehicle or NR-supplemented monocytes in response to LPS (Figure 4E). To directly assess whether the disruption of autophagy blunted LPS-induced type I IFN signaling, pharmacological and genetic modulation of autophagy was performed in primary human monocytes. Interestingly, pharmacological blunting of autophagosome assembly by 3-MA or the disruption of autolysosome formation by nocodazole blunted IFN-β secretion to a similar degree in the vehicle control and NR-supplemented monocytes, whereas the effect of NR in blunting type I IFN production was maintained despite the inhibition of autolysosomal acidification by chloroquine (Figure 4F). In parallel, siRNA targeting resulted in the robust reduction in *ULK1*, *ATG3*, and *ATG5* transcript levels (Supplemental Figure 3A) in parallel with the blunting of IFN-β production in response to LPS. An ameliorative effect of NR in reducing IFN-β production was not uniform; rather, it had an additive effect after ULK1 and ATG3 knockdown but not in response to the depletion of ATG5 (Figure 4G). The abolishment of the NR effect after ATG5 knockdown may reflect the central role of ATG5 in macroautophagy (31) and its essential mediator role in type I IFN production in plasmacytoid DCs (32). Conversely, administration of verapamil, a nonimmunomodulatory activator of autophagy (33), abolished the ability of NR to blunt autophagy and IFN-β production (Figure 4, H and I).

### NR-mediated suppression of type I IFN is NAD^+^ level dependent.

**Figure 5:**
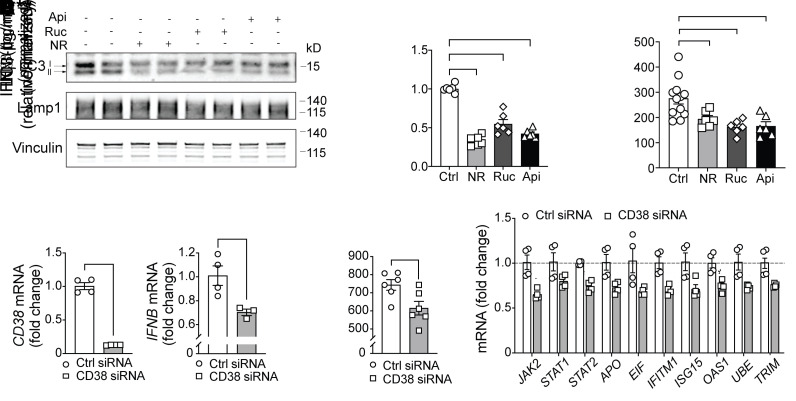
Interventions to increase NAD^+^ levels reduce autophagy and monocytic IFN-β production. (**A**) Representative immunoblot of LC3 (I and II) and Lamp1 in human monocytes preincubated with vehicle or NR and PARP inhibitor rucaparib (Ruc) (10 μM) or CD38 inhibitor apigenin (Api) (20 μM). (**B**) Quantification of LC3-II level in vehicle-, NR-, Ruc-, or Api-treated monocytes normalized to loading controls (*n =* 6, 3 experiments). (**C**) IFN-β production induced by LPS (10 ng/mL for 8 hours) was measured by ELISA from monocytes treated with Ruc or Api for 8 hours (representative results from 3 experiments). (**D**) Relative mRNA expression of *CD38* and *IFNB* from monocytes transfected with either control siRNA or CD38 siRNA (*n =* 3–4 replicates/treatment). (**E**) IFN-β production was measured by ELISA from monocytes transfected with either control siRNA or CD38 siRNA (representative results from 3 experiments). (**F**) Relative mRNA expression of ISGs from monocytes transfected with either control siRNA or CD38 siRNA (*n =* 4 replicates/treatment). Data analyzed using 1-way ANOVA followed by Dunnett’s multiple-comparison test (**B** and **C**) or unpaired 2-tailed Student’s *t* test (**D**–**F**). All data represented as mean ± SEM. *P < 0.05; **P < 0.01; ***P < 0.001; ****P < 0.0001.

The autophagy and immunomodulatory effects of NR could be in part due to its capacity to generate NAD^+^ that could offset bioenergetic and redox stressors that would increase NAD^+^ demand in the context of monocyte or macrophage activation (34, 35). To begin to evaluate this, we assessed the transcript levels of the NAD^+^-consuming family of poly ADP ribose polymerase (PARP) enzymes and of the lymphocyte differentiation antigen and NADase CD38 in primary human monocytes after LPS administration. Within 2 hours of LPS exposure, the *CD38* transcript level was significantly increased, and at 4 hours the levels of numerous transcripts encoding PARP enzymes were similarly induced with the further elevation of *CD38* transcript levels (Supplemental Figure 3B). Interestingly, these are the precise *PARP* genes upregulated by the pattern recognition system and IFN signaling evident in response to coronavirus infection (26). Next, NAD^+^ levels were manipulated using a broad PARP inhibitor (rucaparib) and a pharmacological inhibitor of CD38 (apigenin). The impact of these compounds on autophagy and LPS-induced IFN-β secretion was assessed. Both inhibition of NAD^+^ consumption and breakdown paralleled the effect of NR in blunting autophagy (Figure 5, A and B) and IFN-β secretion (Figure 5C). Furthermore, to validate the role of NAD^+^ levels in type I IFN signaling, *CD38* was depleted using siRNA in primary human monocytes. The extent of knockdown was robust, with an approximately 80% reduction in its transcript level (Figure 5D). Interestingly, depletion of CD38 is known to blunt autophagy during LPS-induced liver injury (36), and here we showed that its knockdown similarly reduced transcript levels of IFN-β (Figure 5D) and blunted LPS-simulated monocytic IFN-β secretion (Figure 5E) as well as genes encoding ISGs (Figure 5F).

### NR mediated suppression of type I IFN is independent of sirtuin knockdown.

The NAD^+^-dependent sirtuin family of deacylase enzymes are proposed to contribute to immunomodulation (37, 38), and NR has been shown to increase deacetylase activity of numerous sirtuin enzymes (39). At the same time, it is predicted that only SIRT1, SIRT3, and SIRT5 have sufficiently high *K_m_* values where NAD^+^ levels may be rate limiting (40). Nevertheless, we generated siRNA targeted knockdown of 5 mammalian family members in primary human monocytes to assay type I IFN signaling in the presence or absence of NR. Sirtuin knockdown efficiency was 60% or greater in all siRNA experiments (Supplemental Figure 3C). However, we found that the blunting effect of NR on IFN-β secretion was not attenuated by the knockdown of these sirtuin enzymes (Supplemental Figure 3D).

### NR-mediated increase in inosine level reduces type I IFN signaling.

**Figure 6:**
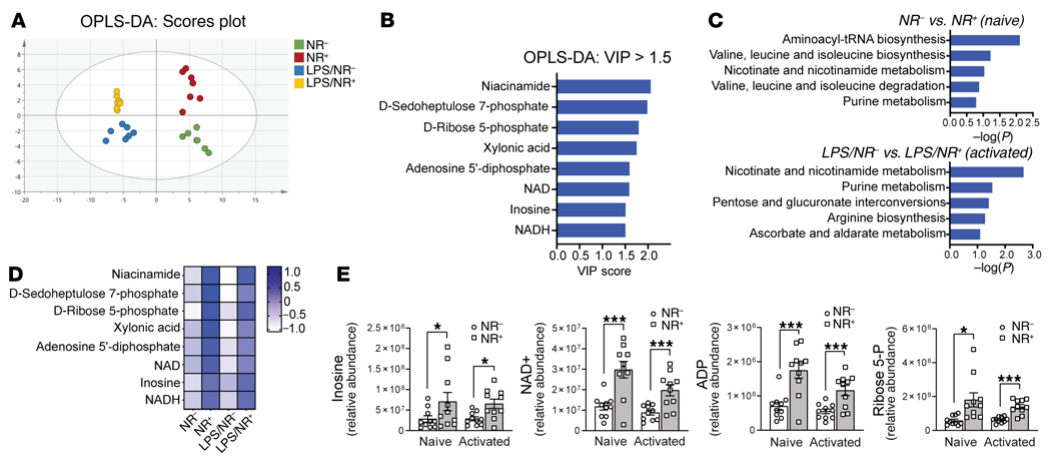
NR incubation increases pentose phosphate pathway and purine intermediates in naive and activated monocytes. (**A**) OPLS-DA score scatterplot showing clear separation between vehicle and NR-supplemented sample clusters at both baseline and in response to LPS stimulation, with no significant outliers (*n =* 7 individuals/group). (**B**) Bar plot of metabolites with VIP score greater than 1.5 that best distinguished between the vehicle control and NR-supplemented groups. (**C**) Pathway enrichment analysis on significantly different metabolites in vehicle control and NR-supplemented groups at baseline (naive) and in response to LPS (activated). (**D**) The heatmap on relative levels of metabolites with VIP score greater than 1.5 in vehicle control and NR-supplemented groups at baseline and in response to LPS activation. (**E**) Relative abundance of inosine, NAD^+^, ADP, and ribose 5-P in vehicle control and NR-supplemented groups. Data analysis of substrate levels (**E**) was performed using unpaired 2-tailed Student’s *t* test. Data represented as mean ± SEM. *P < 0.05; ***P < 0.001.

**Figure 7:**
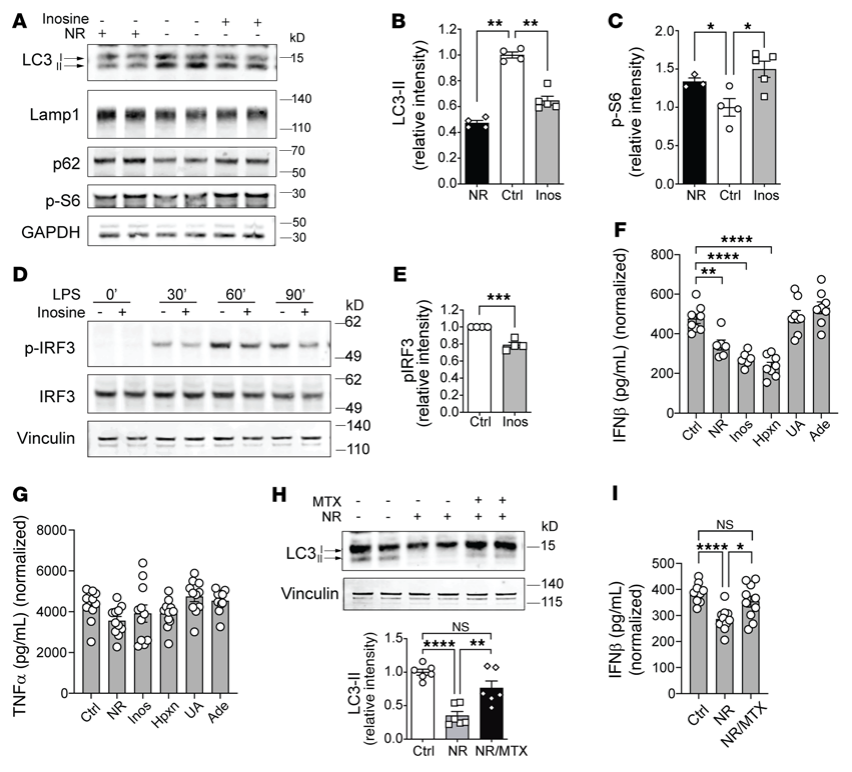
NR-mediated induction of inosine reduces autophagy and monocytic IFN-β production. (**A**) Representative immunoblot of LC3 (I and II), p62, Lamp1, and phospho-S6 in human monocytes preincubated with vehicle or NR or inosine for 1 hour followed by LPS stimulation (10 ng/mL for 5 hours). Quantification of LC3-II (**B**) or phospho-S6 level (**C**) in vehicle-, NR-, or inosine-incubated monocytes normalized to GAPDH level (*n =* 4–5 replicates, 2 experiments). (**D**) Representative immunoblot of phospho-IRF3 level in human monocytes pretreated with vehicle or inosine (0.5 μM) overnight followed by LPS stimulation (10 ng/mL for 30 minutes, 60 minutes, 90 minutes). (**E**) Quantification of phospho-IRF3 level at 30 minutes after LPS stimulation normalized to vinculin level (*n =* 4, 4 experiments). (**F**) IFN-β production and (**G**) TNF-α induced by LPS (10 ng/mL for 7 hours) were measured by ELISA from monocytes incubated overnight with NR, inosine (Inos), hypoxanthine (Hpxn), uric acid (UA), or adenine (Ade) (*n =* 5–10 replicates/treatment; representative results from 2 experiments). (**H**) Representative immunoblot and quantification of LC3 (I and II) in human monocytes incubated overnight with vehicle, NR, or NR + methotrexate (MTX) (*n =* 6, 3 experiments). (**I**) IFN-β production induced by LPS (10 ng/mL for 7 hours) was measured by ELISA from monocytes treated with vehicle control, NR, or NR + MTX overnight (*n =* 10 replicates/treatment; representative results from 2 experiments). Data analysis using 1-way ANOVA followed by Dunnett’s multiple-comparison test (**B**, **C**, **F**, and **G**) or unpaired 2-tailed Student’s *t* test (**E**) or 1-way ANOVA followed by Tukey’s multiple-comparison test (**H** and **I**). All data represented as mean ± SEM. *P < 0.05; **P < 0.01; ***P < 0.001; ****P < 0.0001.

Given that NAD^+^ functions as a coenzyme in a large number of redox-dependent enzymatic reactions, a challenge arising was to delineate which, if any, of these metabolic processes may contribute to the immunomodulatory effect of NR. To narrow down potential metabolic pathways, we employed a targeted metabolomics approach to assay the effect of NR on naive and LPS-stimulated monocyte steady-state metabolite levels. Using orthogonal partial least squared discrimination analysis (OPLS-DA) to model metabolite abundance in vehicle and NR-supplemented naive and activated monocytes, we identified the metabolites that best distinguished between vehicle control and NR-supplemented groups as those candidates with a variables of prediction score greater than 1.5. The OPLS-DA score scatterplot showed clear separation between NR and vehicle control sample clusters, both at baseline and in response to LPS stimulation, with no significant outliers (Figure 6A). Unsurprisingly, NAD^+^ metabolic intermediates predominated, and other NR-responsive metabolites in monocytes included changes in the steady-state levels of intermediates in the PPP and in purine metabolism (Figure 6B). Subsequent pathway analysis identified purine metabolism as the most highly significantly distinguishing pathway following the expected role of changes in nicotinate and nicotinamide metabolism in naive and LPS-activated monocytes (Figure 6C). The heatmap of relative levels of NAD^+^, PPP, and purine pathway metabolites showed the similar NR induction of these metabolites at baseline and in response to LPS (Figure 6D). The relative abundance of these metabolites is shown in Figure 6E and Supplemental Figure 4. Given that the purine intermediate inosine confers antiinflammatory effects (41) and inosine levels function as a nutrient sensor for the autophagy mediator mTORC1 (42), we explored the effect of inosine supplementation on autophagy and type I IFN secretion in monocytes. The supplementation of LPS-activated monocytes with inosine blunted autophagy and increased phosphorylation of the mTORC1 substrate S6 (Figure 7, A–C). In parallel, inosine supplementation blunted the temporal phosphorylation of IRF-3 in response to LPS stimulation (Figure 7, D and E). These effects of inosine similarly blunted LPS-stimulated IFN-β release (Figure 7F). Interestingly, hypoxanthine, the salvage pathway intermediate for inosine production, mirrored the effect of inosine in blunting INF-β secretion, whereas adenosine and uric acid did not (Figure 7F). The selective immunomodulatory effects of inosine and hypoxanthine were exemplified in that they had no effect on TNF-α release in response to LPS (Figure 7G). Interestingly, in the context of LPS monocytic activation, the purine synthesis inhibitor methotrexate (43) attenuated the NR-mediated blunting of autophagy (Figure 7H) and IFN-β secretion (Figure 7I).

### NR blunts LPS-induced type 1 IFN production in healthy participants and patients with SLE.

**Figure 8:**
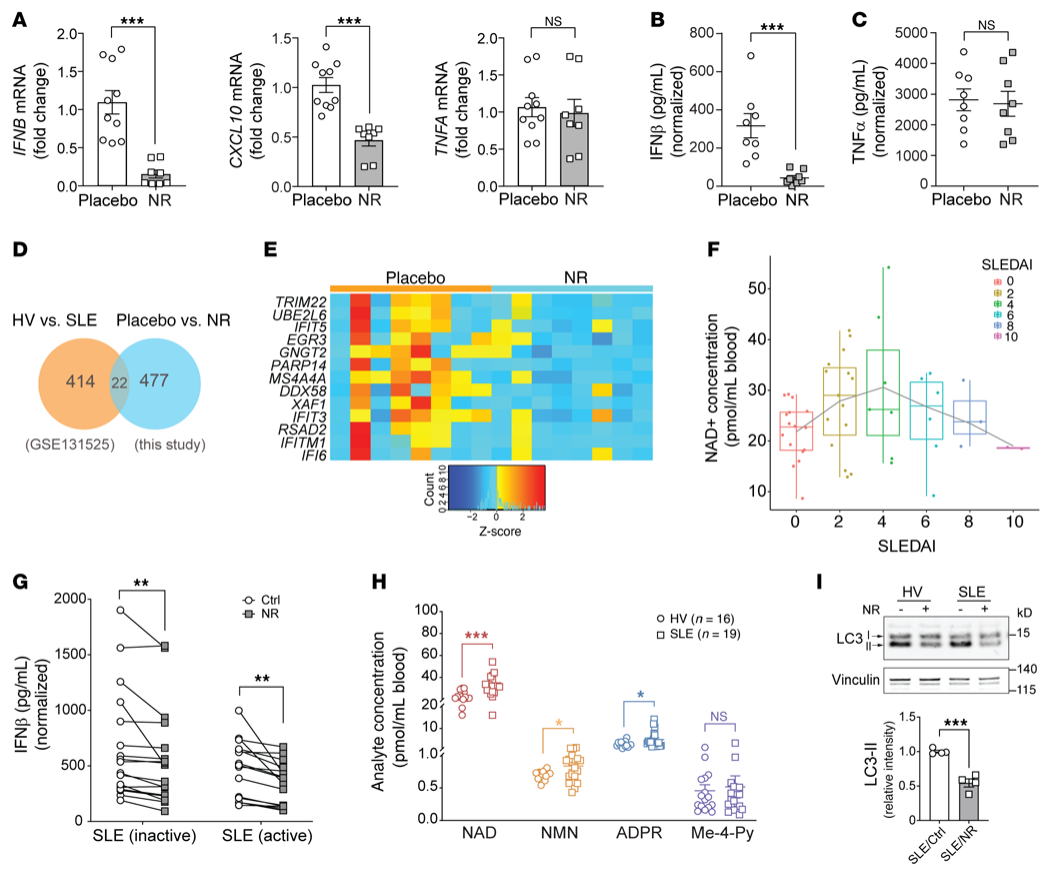
NR blunts monocytic type I IFN in healthy volunteers and in monocytes from patients with SLE. (**A**) Relative mRNA expression of *IFNB*, *CXCL10,* and *TNFA* in cryopreserved monocytes from healthy volunteers exposed to the placebo or NR for 7 days (*n =* 8–10 individuals/group). (**B**) IFN-β and (**C**) TNF-α production measured by ELISA in parallel from cryopreserved monocytes described in Figure 7A (*n =* 8 individuals/group). (**D**) Venn diagram showing 22 overlapping DE genes between controls versus SLE (GSE131525) and placebo versus NR comparison (this study). (**E**) Heatmap on relative levels of 13 overlapping DE genes that were downregulated by NR. (**F**) A box plot of NAD^+^ concentration for the indicated SLEDAI score on the *x* axis is depicted. The dot/circle on each box plot represents individual NAD^+^ measurement for the indicated SLEDAI score. A trend line was generated using LOESS (locally estimated scatter plot smoothing). (**G**) IFN-β production induced by LPS (10 ng/mL for 6 hours) was measured by ELISA in monocytes from patients with SLE with different disease activity (inactive: SLEDAI ≤4; active: SLEDAI >4) that incubated with vehicle or NR for 16 hours (*n =* 17 for SLE inactive group; *n =* 16 for SLE active group). (**H**) Levels of NAD^+^, NMN, and ADPR from whole blood of healthy volunteers (HV) and patients with SLE measured by LC-MS (*n =* 16 for HV group; *n =* 19 for SLE group). (**I**) Representative immunoblot and quantification of LC3-II in SLE monocytes incubated with vehicle or NR for 16 hours and then stimulated with LPS (10 ng/mL) for 2 hours. Data were analyzed by unpaired 2-tailed Student’s *t* test (**A**–**C**, **H**, and **I**) or paired 2-tailed Student’s *t* test (**G**). All data represented as mean ± SEM. *P < 0.05; **P < 0.01; ***P < 0.001.

To assess whether these mechanisms were operational in the human study, we resuspended cryopreserved PBMCs and isolated and cultured monocytes from the placebo and NR-supplemented study groups. Consistent with the ex vivo NR administration effects, the prior in vivo supplementation with NR blunted steady-state transcript levels of genes encoding IFN-β and its responsive chemokine *CXCL10*, while the *TNF-α* transcript level remained unchanged after LPS stimulation (Figure 8A). In parallel, LPS-stimulated monocytes exposed to NR in vivo showed blunted release of IFN-β with no effect on TNF-α secretion (Figure 8, B and C). These data demonstrated that NR supplementation in individuals sufficiently recapitulated the NR type I IFN effects elucidated in primary monocyte cultures (24).

Since SLE is a type I IFN–linked disease, we wanted to explore the common IFN signaling genes that are perturbed in SLE and are also downregulated by NR. For this purpose, we utilized publicly available RNA-Seq data from GEO GSE131525, composed of sequencing data from monocytes isolated from healthy controls and patients with SLE. Differential gene expression analysis identified a large subset of DE genes that were enriched in the type I IFN signaling pathways. As expected, several of these DE genes were upregulated in SLE monocytes, supporting an upregulation of type I IFN in SLE (Supplemental Table 5). DE genes identified from this SLE GEO data set were then compared with the activated placebo versus NR supplementation DE genes in this study and revealed a subset of 22 overlapping genes (Figure 8D). Of those 22 genes, 13 were elevated in SLE (Supplemental Table 5) and blunted by NR (Supplemental Table 6) that included genes linked to NAD^+^ consumption (*PARP14*), innate immunity and autophagy (tripartite motif family protein 22 [*TRIMM22*]), the type I IFN pathway (IFN-induced protein with tetratricopeptide repeats [*IFIT* genes]), XIAP-associated factor 1 (*XAF1*), and the antiviral innate immune response receptor RIG-1 (*DDX58*) (Figure 8E).

To explore this interaction more directly, we extracted monocytes from patients with lupus with various degrees of disease activity as measured by the Systemic Lupus Erythematosus Disease Activity Index (SLEDAI) (44). The study participants’ characteristics and immunosuppressive therapy are shown in Supplemental Tables 7 and 8. Interestingly, SLE has been shown to evoke NAD^+^ depletion by induction of CD38 levels in monocyte and in cytotoxic CD8^+^ T cell populations, where levels in monocytes correlated with disease activity (45) and where higher CD38 levels in SLE cytotoxic CD8^+^ T cells were associated with an impaired capacity to control infections (46). Given these data, we first explored the baseline NAD^+^ levels in whole blood from this population. Our cohort included individuals with SLEDAI scores ranging from 0 to 10 that represent inactive (index ≤4) to mild to moderate disease (index >4). Interestingly, a bimodal distribution of NAD^+^ levels was found, with peak levels at a score of 4, with a relatively symmetrical distribution of steady-state levels across activity scores (Figure 8F). We then evaluated IFN-β secretion in response to LPS in these monocytes, comparing individuals with SLEDAI scores 4 or less and higher than 4, and found that both cohorts were equally responsive to IFN-β blunting by NR (Figure 8G). To begin to explore the mechanism of higher NAD^+^ levels with inactive disease, we assessed NAD^+^ metabolic intermediates (28) in the inactive group (index ≤4) compared with an age- and gender-matched control group. Here, we showed increased NAD^+^ and NMN levels as well as evidence of increased NAD^+^ consumption as implicated by increased levels of ADP ribose (ADPR) moieties of NAD^+^ in the inactive lupus cohort (Figure 8H). In parallel, transcript levels for *IDO1*, the first and rate-limiting enzyme for NAD^+^ de novo synthesis, and of *CD38*, *PARP9*, and *PARP10*, which promote NAD^+^ breakdown and consumption, were concordantly upregulated in the inactive SLE cohort monocytes as compared with healthy volunteers (Supplemental Figure 5). Together, these data suggest that both NAD^+^ production and consumption are activated in early or inactive disease, potentially as a component of intracellular monocytic antiinflammatory programing. This concept warrants further exploration. Finally, we assayed whether NR similarly blunted LC3-II lipidation in individuals with SLE, and found that here NR phenocopied the blunting of the autophagy signature in monocytes from individuals with lupus (Figure 8I).

## Discussion

Although there is growing evidence that NAD^+^ precursors confer antiinflammatory effects, the regulatory mechanisms remain incompletely characterized. This study demonstrated that NR supplementation blunted LPS-induced type I IFN signaling, an important myeloid regulatory program, through increased NAD^+^ levels, the elevation of inosine, and the subsequent suppression of autophagy. These NR-induced effects were found to be operational in monocytes from healthy human volunteers and in monocytes from individuals with SLE, a systemic autoimmune disease characterized by enhanced type I IFN synthesis and a robust type I IFN gene signature (47).

The role of NAD^+^ in monocyte/macrophage biology is probably context and signaling pathway specific. An example is that reduction in de novo synthesis of NAD^+^ in macrophages impairs phagocytosis and resolution of inflammation, with augmentation of macrophage reparative function by NAD^+^ restoration (35). Furthermore, nicotinamide supplementation reduces inflammatory cytokines in macrophages during differentiation and polarization into reparative macrophages (48). In contrast, the activation of murine macrophages by LPS required an intact NAD^+^ salvage pathway to sustain aerobic glycolysis and canonical IL-1β–, TNF-, and IL-6–orchestrated inflammation (34). Our study found that NR-mediated elevation of NAD^+^ levels attenuated the type I IFN signaling pathway in human monocytes and demonstrated this effect in vivo on a young and healthy population as well as ex vivo on middle-aged individuals in the lupus and control groups.

Autophagy is an integral component of type I IFN–mediated viral clearance and can blunt type I IFN signaling via the sequestration and degradation of viral nucleic acids (49). Furthermore, the autophagic vesicle formation protein subunit Atg5 functions as an essential mediator of type I IFN production in plasmacytoid DCs in response to vesicular stomatitis viral infection (32). Additionally, IFN-α production in response to host DNA in autoimmune disease is dependent on the convergence of phagocytic and noncanonical autophagic pathways via LC3-associated phagocytosis (50). Together, these data suggest complex, context-specific crosstalk between the regulation of autophagy and type I IFN signaling. In parallel, LPS has been found to initiate autophagy in different cell types (36, 51). Interestingly, LPS induction of autophagy signals via the TLR4 signaling pathway, which is dependent on TRIF (toll-interleukin-1 receptor domain-containing adaptor-inducing IFN-β) and independent of MyD88 (myeloid differentiation factor 88) (52). Meanwhile, the activation of TBK1 is important in autophagosome formation and in the stabilization of the lipidated states of autophagy mediators (53). Hence, the role of NR in blunting TBK1 phosphorylation and LPS-induced type I IFN (via TRIF signaling) highlights the parallel pathways for autophagy induction and type I IFN signaling and supports the concordance effect of NR in blunting these pathways.

Autophagy is a nutrient- and redox stress–sensing intracellular quality control program. NAD^+^ functions as a coenzyme in numerous redox reactions, so the effect of NR on metabolism could play myriad roles in blunting autophagy. This could be, in part, via modifying metabolite levels and/or by altering reductive equivalents, such as NADPH, to modify oxidative stress susceptibility. Targeted metabolomics was performed on naive and LPS-stimulated primary monocytes after NR exposure to evaluate whether changes in metabolites may be operational in this biology. Numerous PPP and purine salvage pathway intermediates were increased by NR in both the basal state and in response to LPS. Interestingly, the levels of whole-cell NADP^+^ and NADPH were proportionally increased by NR with no change in their ratio. This argues against a change in the redox balance in the blunting of autophagy. Meanwhile, the other PPP intermediates D-sedoheptulose 7-phosphate and ribose-5-phosphate are downstream of PPP oxidoreductase enzymes. Ribose-5-phosphate also functions as a precursor for nucleotide biosynthesis and additional purine salvage pathway intermediates, including inosine and adenosine-5-diphosphate, which were similarly increased by NR. The elevation of the steady-state levels of these intermediates could be compatible with the combination of increased redox enzyme activities as well as a downstream bottleneck of the metabolism of these intermediates. This biochemistry requires further exploration. At the same time, although inosine confers antiinflammatory effects and blunts endotoxemia in mice (54), its role in type I IFN signaling does not appear to have been previously explored. Here, we showed that inosine replicated NR effects on inhibiting autophagy and by blunting IFN-β production. The mechanisms of action of inosine have not been extensively explored but confer protection through adenosine receptor signaling (41) and by the generation of ATP to protect against nutrient-depletion stressors (55). Inosine also appears to preferentially activate ERK signaling (56), which in turn has context-specific modulatory effects on autophagy (57, 58). Although not studied here, these mechanisms may play a role in the modulation of autophagy in LPS-stimulated monocytes and warrant further exploration. At the same time, the role of pharmacological disruption of purine metabolism on immune function is complex, given that methotrexate, which we showed augmented LPS-mediated autophagy and type I IFN, exhibits clinical immunosuppressive effects. Methotrexate’s immunosuppressive action is multifactorial (59, 60), although interestingly, one of the modes of action blunts polyamine production, and spermidine is known to trigger autophagy (61). Hence, further studies will be required to reconcile findings of methotrexate on NR effects on TLR4-triggered monocytic type I IFN versus its broader immunosuppressive functions. Furthermore, given the myriad immunosuppressive agents employed to treat SLE, a reductionist approach would need to be undertaken to understand how different agents and their dosing modulate both NAD^+^ biology and autophagy.

The role of NAD^+^ metabolism in monocytes in SLE has not been extensively examined. However, the levels of the CD38 receptor, which functions as an NADase, is elevated in T cells of patients with lupus with increased disease activity, and the stimulation with CD38 antibodies results in the production of Th1 and Th2 cytokines (62). More recent evidence shows that elevated CD38 levels in CD8^+^ T cells from individuals with SLE reduces CD8^+^ T cell cytotoxicity (46). Interestingly, this effect was found to be dependent on reduced activity of SIRT1, with subsequent epigenetic effects, and increased NAD^+^ degradation in CD8^+^ T cells was postulated to contribute to the known susceptibility to infections in patients with SLE (46). Moreover, CD4^+^ and CD8^+^ T cells from individuals with elevated type I IFN show elevated NAD^+^ degradation enzymes, including CD38 and PARPs 9, 10, and 12 (63). Furthermore, administration of IFN-α to CD8^+^ T cells reduced the NAD^+^/NADH ratio and the in vitro administration of nicotinamide mononucleotide augmented the NAD^+^/NADH ratio in CD8^+^ T cells (63). Combining our findings with the effects on different T cell populations suggest that the perturbation of NAD^+^ metabolism may have more extensive effects on the pathogenesis of SLE. Furthermore, given that GWAS supports a role of autophagy in lupus where autophagy is induced in B and T cells (64), the finding that NR blunts this program in monocytes from patients with SLE suggests that NR may have broader effects on SLE immunomodulation.

Finally, the beneficial effects of NAD^+^ precursor supplementation in individuals are most well-established for nicotinic acid and nicotinamide (65). In contrast, although NR has been found to be safe to administer in people, its effect on systemic metabolic perturbations in improving insulin sensitivity has been questioned (66), and whether this salvage pathway intermediate has a benefit in vivo and/or in any particular organ system remains unknown (65). In this study, we demonstrated that NR supplementation did modulate type I IFN signaling and appeared to be operational in individuals’ monocytes.

Limitations in this study include that this was a pilot study and it would need to be repeated in a larger cohort in vivo where NR should be administered to individuals with SLE to evaluate its effect in the context of their complex therapeutic regimens. At the same time, in this randomized pilot study, intergroup differences were evident, including differences in lymphocyte number at baseline and fasting levels of glucose, insulin, and monocyte numbers (Supplemental Tables 1 and 2). These differences were no longer evident in the refed state when the analysis was performed; nevertheless, whether these baseline and fasting intergroup differences had an effect on the data needs further validation. Additionally, when the study was designed to assay the monocytic effects of NR at the transcript level (RNA-Seq), LPS was included as the inflammatory trigger, and subsequent analysis uncovered that type I IFN signaling was a prominent NR-modulated pathway. Further in vivo studies will be necessary to assess whether NR modulates cGAS/STING-mediated type I IFN signaling, which is implicated in autoimmune diseases, including SLE (67). We also recognize that recent evidence shows that fasting modulates bone marrow sequestration of monocytes, and whether this plays any role in subsequence transcriptional profile of monocytes in response to refeeding and NR remains unknown. A technical limitation in studying this biology is the low abundance of monocytes in circulation, which limits the number of cells that can be isolated from the restricted amount of blood that can be drawn from individuals.

In conclusion, this study showed that NR supplementation blunted type I IFN activation in human monocytes acutely exposed to LPS. This immunomodulatory effect of NR functions in part via the restoration of NAD^+^ levels, through inosine signaling and by blunting autophagy in monocytic cells. Furthermore, this regulatory effect is operational in monocytes isolated from patients with SLE that intrinsically exhibit evidence of enhanced basal type I IFN signaling. Finally, this study raises the question as to whether this NAD^+^ precursor (68) may be a useful adjunctive supplement in patients with SLE, as dysregulation of both autophagy and the type I IFN pathway play crucial pathogenic roles in this disease.

## Methods

### Study design and participants.

This pilot study was performed at the NIH Clinical Center on 35 individuals with an average age of 24 years and average BMI of 24. Healthy volunteers were screened prior to signing consent, and study participants were then randomly assigned to a 7-day supplementation of either NR (1000 mg daily) or a matching placebo. After 1 week of therapy, study participants underwent a 24-hour fast followed by an early morning, fixed 500-calorie meal. Blood draw was taken before and 3 hours after feeding. Participants had a choice between 2 isocaloric breakfasts: (a) vegetable omelet, toast with butter and jelly, and orange juice; or (b) oatmeal with walnuts, brown sugar, dried cranberries, and milk. The schematic of the blood draw protocol and immune function studies is shown in Figure 1A. The study participants’ serum insulin, glucose, and growth hormone levels at the end of the 24-hour fast and 3 hours after the fixed-calorie meal are shown in Supplemental Figure 1B and Supplemental Table 1.

All patients with SLE (*n =* 60) fulfilled the revised American College of Rheumatology classification criteria (69). As controls, age- and sex-matched healthy volunteers were recruited (*n =* 23) through the healthy blood donor population at the NIH Clinical Center Blood Bank. The average age for the patients with SLE was 48 years and for the healthy donors was 46 years. All study participants were females except 3 males with SLE who were included in the whole-blood NAD^+^ measurement. The disease activity score for the patients with SLE was measured using the SLEDAI scoring system (44). The study participants’ demographics, range of SLEDAI scores, and the therapy being administered are described in Supplemental Table 8.

### Human monocyte cultures.

Primary PBMCs were isolated from human blood by density centrifugation using Lymphocyte Separation Medium (MP Biomedicals). Monocytes were negatively selected (monocyte isolation kit from Miltenyi Biotec). Monocytes were then plated 0.15 × 10^6^/well onto 96-well plate (for ELISA) or 10^6^/well onto 12-well plates (for RNA isolation or Western blotting) in RPMI media supplemented with 10% human serum. Elutriated monocytes were obtained from the blood bank of NIH.

### RNA-Seq and bioinformatics analysis.

Total RNA was extracted with the NucleoSpin RNA kit (Takara), and RNA quality was assessed by Agilent Bioanalyzer. Libraries were prepared using TruSeq stranded mRNA kit (Illumina) and sequenced in a HISeq 3000 (Illumina) by the DNA Sequencing and Genomics Core at NHLBI. FastQC (http://www.bioinformatics.babraham.ac.uk/projects/fastqc) was used to confirm the quality of RNA-Seq FASTA files. Adaptor trimming, RNA-Seq alignment, PCA, and differential pathway analysis were performed as previously described (20). Genes with *P* value less than 0.05 were considered DE.

### Inhibitors, siRNA, and nucleofection of human monocytes.

NR (ChromaDex) cell incubation (0.5 mM) for 16 hours was used prior to the subsequent assays. PARP inhibitor rucaparib camsylate (Sigma-Aldrich) and CD38 inhibitor apigenin (MilliporeSigma) were used in cell culture at 10 μM or 20 μM for 8 hours, respectively. Autophagy-related inhibitors or activator were used in the following concentrations for overnight incubation with the cells: 3-MA (5 mM), nocodazole (1 μg/mL), chloroquine (10 μM), and verapamil (50 μM). Primary human monocytes were incubated with purine metabolism–related chemicals at the following concentrations overnight before LPS stimulation: inosine (0.5–5 μM), hypoxanthine (5 μM), uric acid (5 μM), adenine (5 μM), and methotrexate (50–100 μM). ON-TARGET*plus* siRNA for gene knockdown was utilized targeting CD38, sirtuins (Sirt1, Sirt2, Sirt3, Sirt5, and Sirt7), and autophagy proteins (ULK1, ATG3, ATG5) (Dharmacon, Horizon Discovery). siRNA was incubated with a mixture of nucleofection solution (Human Monocytes Nucleofector kit; Lonza) and primary human monocytes and placed in nucleofection cuvettes subjected to program Y-010 for the Nucleofector 2b device (Lonza). After nucleofections, 500 μL RPMI medium was immediately added into cuvettes. Cells were then plated in a 96-well plate or 12-well plate and incubated at 37°C under 5% CO_2_ for 36 hours before assays.

### RNA isolation and qPCR analysis.

Total RNA was extracted from monocytes (NucleoSpin RNA kit, Takara) and quantified (NanoDrop Spectrophotometer, Thermo Fisher Scientific). cDNA was synthesized with the SuperScript III First-Strand Synthesis System for RT-PCR (Thermo Fisher Scientific). Real-time qPCR was performed using FastStart Universal SYBR Green Master (Rox) (Roche Holding) and run on LightCycler 96 Systems (Roche Holding). Transcript levels of IFN-β, *TNF-α*, *CXCL10*, and *18s* rRNA were measured using validated gene-specific primers (QIAGEN). Primers for ISGs were custom-synthesized at IDT Inc. Relative gene expression was quantified by normalizing cycle threshold values with 18S rRNA using the 2^–ΔΔCt^ cycle threshold method.

### Western blotting.

Human monocytes were lysed using RIPA buffer supplemented with protease inhibitor cocktail (Roche) and phosphatase inhibitors (Sigma-Aldrich). Lysates were separated by NuPAGE 4%–12% Bis-Tris Protein Gels (Thermo Fisher Scientific) and transferred to nitrocellulose membranes (Trans-Blot Turbo Transfer System (Bio-Rad). Membranes were blocked with 50% Odyssey Blocking buffer in PBS-T (0.1% Tween 20 in PBS) and incubated with appropriate antibodies overnight at 4°C. The following antibodies were used: STAT1, phospho-STAT1(Y701), STAT2, phospho-STAT2 (Y690), TBK1, phospho-TBK1 (Ser172), LAMP1 (D2D11) XP, SQSTM1/p62, phospho-S6 (Ser235/236), GAPDH, and β-actin (Cell Signaling Technology); IRF3 and phospho-IRF3 (S386) (Abcam); and LC3 and vinculin (Sigma-Aldrich). Antibody suppliers and catalog numbers are shown in Supplemental Table 10. Primary antibody incubations were followed by incubation with IRDye secondary antibodies for 1 hour at room temperature. Immunoblots were imaged using Odyssey CLx Imaging System (LI-COR Biosciences). Protein band intensity was quantified using ImageJ (NIH) and normalized to β-actin or vinculin. Detailed information about antibodies used in this study is summarized in Supplemental Table 10.

### Cell stimulation and cytokine/chemokine assays.

Human monocytes were incubated at 1.5 × 10^6^ cells per mL in a 96-well plate in complete RPMI medium (25 mM HEPES plus 10% human serum in RPMI medium) and primed with 100 ng/mL human IFN-γ (R&D Systems) for 16 hours before stimulations with 10 ng/mL LPS (Ultrapure Salmonella Minnesota R595; Enzo Life Sciences) for 6–8 hours. Cell culture supernatants were collected, centrifuged to remove cells and debris, and stored at –80°C for later analysis. Cytokines of human IFN-α2, IFN-β, and TNF-α and chemokine CXCL10 were assayed by ELISA (R&D Systems). Results were normalized to cell number as determined by the CyQuant cell proliferation assay (Invitrogen).

### LC-MS measurement of NAD^+^ and related analytes.

First, 100 μL snap-frozen whole blood from each study participant and reconstitution immediately prior to LC-MS analysis (28). Internal standards were stable isotope analogs of nucleotides and nucleosides labeled with ^13^C glucose. NAAD was the only nucleotide of interest not having a labeled analog; ^13^C_10_ NAD was used as its internal standard. d3-methyl-4-pyridone-3-carboxamide was used as internal standard for methyl-4-pyridone-3-carboxamide. A Waters Acquity TQD liquid chromatography-mass spectrometer (LC-MS) was used to separate and quantify analytes. Nucleotides were determined using a 2.1 × 100 mm Hypercarb analytical column (Thermo Fisher Scientific) with a mobile phase of (a) 7.5 mM ammonium acetate + 0.05% NH_4_OH and (b) acetonitrile + 0.05% NH_4_OH. A gradient elution was used with analytes eluting from 4 to 10 minutes. The total run time was 19 minutes.

### Metabolomics and data analysis.

Human monocyte cultures were supplemented with 0.5 mM NR or vehicle control for 24 hours without LPS stimulation (naive monocytes) or with 1 ng/mL LPS for 1 hour (activated monocytes). Cells were the scraped off the plates and centrifuged at 4°C for 10 minutes, and cell pellets were collected and snap-frozen in liquid nitrogen. Frozen pellets were thawed at 4°C for an hour, and 0.5 mL of cold 80:20 methanol/water was added for protein precipitation. The methanol/water mixture contained 13C-glucose and 13C-glutamic acids as reference standards. Supernatants were dried and reconstituted in LC-matching solvent containing 13C-tyrosine and 13C-lactate reference standards to monitor the LC-MS assay performance. Targeted LC-MS metabolite analysis was performed on a duplex LC-MS system composed of 2 Shimadzu UPLC pumps, CTC Analytics PAL HTC-xt temperature-controlled auto-sampler, and AB Sciex 6500+ Triple Quadrupole MS equipped with electrospray ionization source. UPLC pumps were connected to the auto-sampler in parallel and were able to perform 2 chromatography separations independently from each other. Each sample was injected twice on 2 identical analytical columns (Waters XBridge BEH Amide XP) performing separations in hydrophilic interaction LC (HILIC) mode. MS data acquisition was performed in multiple reaction–monitoring (MRM) mode. The LC-MS system was controlled using AB Sciex Analyst 1.6.3 software. The LC-MS assay targeted 363 metabolites and 4 spiked stable isotope-labeled internal standards (SILISs). Measured MS peaks were integrated using AB Sciex MultiQuant 3.0.3 software. In addition to the study samples, 2 sets of quality controls (QCs) were used to monitor the assay performance as well as data reproducibility. QC(S) was a pooled human serum sample used to monitor the assay performance and QC(I) was pooled study samples (pooled after sample preparation) used to monitor data reproducibility. Median coefficient of variation (CV) was 7.70% and 8.45% for QC(I) and QC(S), respectively, for the monocyte extracts. Metabolite abundance was normalized to protein content. Multivariate modeling was performed using Simca software (version 15, Umetrics), and pathway analysis was performed using MetaboAnalyst 5.0 (metaboanalyst.ca).

### Data and materials availability.

All data associated with this study are present in the paper or supplemental material. The mRNA-Seq data sets can be accessed at GEO GSE192536 (https://www.ncbi.nlm.nih.gov/geo/query/acc.cgi?acc=GSE192536). 

### Statistics.

Statistical analysis was performed using Prism 7 software (GraphPad) and results are represented as mean ± SEM unless otherwise indicated. Comparisons of 2 groups were calculated using a paired or unpaired 2-tailed Student’s *t* test. Comparisons of more than 2 groups were calculated using 1-way ANOVA followed by a multiple-comparison test (Dunnett’s or Tukey’s). Two-way ANOVA was used if there were 2 independent variables. For all tests, a *P* value less than 0.05 was considered significant.

### Study approval.

To study the effect of NR on monocytes, healthy volunteers were recruited at the NIH Clinical Center and provided consent on an IRB-approved protocol (NHLBI Study to Evaluate the Effect of Nicotinamide Riboside on Immunity, ClinicalTrials.gov NCT02812238). The blood from healthy volunteers for functional validation studies in monocytes was obtained from individuals who consented to and enrolled in the NIH Clinical Center blood bank protocol (ClinicalTrials.gov NCT00001846). Patients with SLE were similarly recruited and provided informed written consent on an IRB-approved protocol (NIAMS SLE natural history protocol, ClinicalTrials.gov NCT00001372).

## Author contributions

JW, KS, and MNS conceived the project. MNS and MJK secured funding. JW, KS, LPB, CB, MJK, MP, and MNS designed the experiments. RDH and LPB recruited study participants. JW, KS, AL, AMM, VS, MB, SH, RT, MSS, KW, and LPB carried out experiments. JW, KS, SH, AMM, VS, CB, MJK, MP, KW, and MNS analyzed data. JW, KS, and MNS wrote the manuscript. CB, RT, KW, and MJK edited the manuscript.

## Supplementary Material

Supplemental data

ICMJE disclosure forms

Supplemental table 3

Supplemental table 4

Supplemental table 5

Supplemental table 6

Supplemental table 8

## Version 1 01/13/2022

In-Press Preview

## Version 2 03/01/2022

Electronic publication
