# Supplementary material for: Boosting NAD+ blunts TLR4-induced type I IFN in control and systemic lupus erythematosus monocytes
Source: J Clin Invest. 2022 Mar 1;132(5):e139828. doi: 10.1172/JCI139828 (PMC8884917; doi:10.1172/JCI139828)
Supplement: Supplemental data [file jci-132-139828-s087.pdf]

Supplemental Material

Boosting NAD$^+$ blunts toll-like receptor-4 induced type-I interferon in control and systemic lupus erythematosus monocytes

Jing Wu[1], Komudi Singh[1,2,], Amy Lin[1], Allison M. Meadows[1], Kaiyuan Wu[1], Vivian Shing[1], Maximilian Bley[1], Shahin Hassanzadeh[1], Rebecca D. Huffstutler[3], Mark S. Schmidt[4], Luz P. Blanco[5], Rong Tian[6], Charles Brenner[4,7], Mariana J. Kaplan[5], Mehdi Pirooznia[2] and Michael N. Sack[1,3*]

Supplemental Figures

Supplemental Figure 1. Flow diagram, blood biochemical assay and pipeline for RNAseq analysis.

Supplemental Figure 2. Bioinformatic characterization of RNA sequencing conducted in human monocytes following *in vivo* NR supplementation.

Supplemental Figure 3. LPS increases CD38/PARPs mRNA levels and Sirtuin depletion did not modulate NR-mediated attenuation of IFN β release induced by LPS.

Supplemental Figure 4. Multiple metabolites involved in PPP, nicotinamide metabolism and Purine Metabolism are upregulated in NR supplemented groups.

Supplemental Figure 5. Relative mRNA expression of enzymes involved in *de novo* NAD$^+$ synthesis and NAD$^+$ consumption is upregulated in monocytes from inactive Systemic Lupus Erythematosus patients (SLE).

Supplemental Tables

Supplemental Table 1. Research subjects' characteristics and laboratory results under baseline, fasted and refed states with Placebo or NR supplementation.

Supplemental Table 2. Complete Blood Count in baseline, fasted and refed states in human subjects with Placebo or NR supplementation.

Supplemental Table 7. Characteristics of Healthy Volunteers and Systemic Lupus Erythematosus patients enrolled in the study.

Supplemental Table 9. Customer Synthesized Primers for qRT-PCR.

Supplemental Table 10. Wetern Blotting Antibodies Used in this study.

Other Supplemental Tables (Microsoft Excel format)

Supplemental Table 3. Differentially expressed genes comparing NR supplemented vs placebo supplemented naïve monocytes.

Supplemental Table 4. Differentially expressed genes comparing NR supplemented vs placebo supplemented LPS-activated monocytes.

Supplemental Table 5. Upregulated overlapping genes comparing SLE vs HV monocytes from GSE131525.

Supplemental Table 6. Downregulated overlapping genes comparing NR supplemented vs placebo supplemented monocytes *in vivo*.

Supplemental Table 8. Characteristics and therapy being administered in Systemic Lupus Erythematosus patients enrolled in the study.

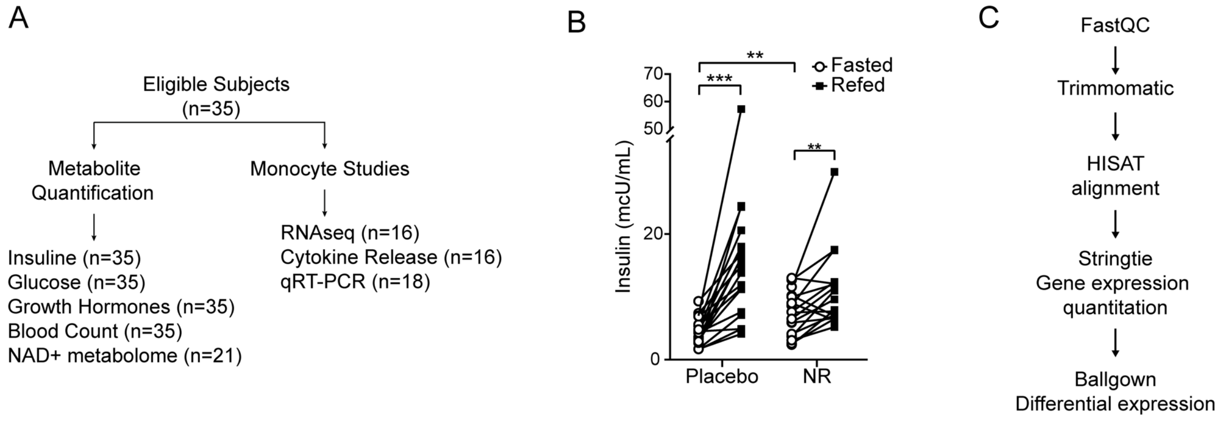

Supplemental Figure 1. Flow diagram, blood biochemical assay and pipeline for RNAseq analysis.

(A) Flow diagram of NR clinical study in healthy subjects. (B) Subjects' sera insulin levels were measured at the end of the 24-hour fast (Fasted) and 3 hours following the fixed caloric meal (Refed) (n = 18 for placebo group; n=17 for NR group). Paired (fasted vs. refed) or unpaired (NR vs. placebo) two-tailed Student's *t*-test. Data were represented as mean ± SEM. **p*<0.05; ***p*<0.01; ****p*<0.001; ns, not significant. (C) The pipeline to analyze the fasta files from RNA sequencing conducted in monocytes from healthy volunteers administrated with Placebo or NR (1000mg daily) for 7 days.

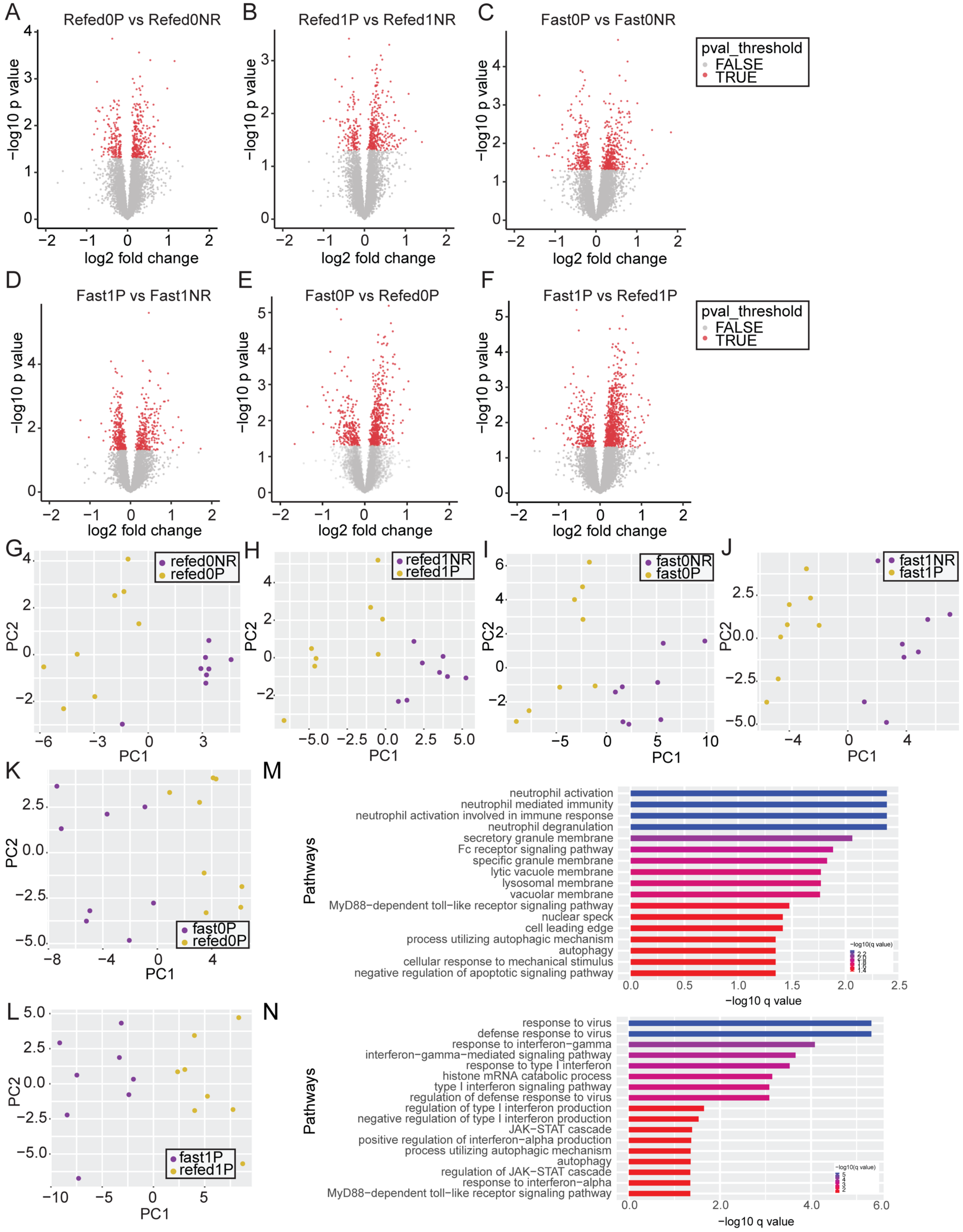
A
Refed0P vs Refed0NR
B
Refed1P vs Refed1NR
C
Fast0P vs Fast0NR
pval_threshold
FALSE
TRUE
D
Fast1P vs Fast1NR
E
Fast0P vs Refed0P
F
Fast1P vs Refed1P
−log10 p value
log2 fold change
G
refed0NR
refed0P
H
refed1NR
refed1P
I
fast0NR
fast0P
J
fast1NR
fast1P
K
fast0P
refed0P
L
fast1P
refed1P
PC1
PC2
M
Pathways
neutrophil activation
neutrophil mediated immunity
neutrophil activation involved in immune response
neutrophil degranulation
secretory granule membrane
Fc receptor signaling pathway
specific granule membrane
lytic vacuole membrane
lysosomal membrane
vacuolar membrane
MyD88−dependent toll−like receptor signaling pathway
nuclear speck
cell leading edge
process utilizing autophagic mechanism
autophagy
cellular response to mechanical stimulus
negative regulation of apoptotic signaling pathway
−log10 q value
N
Pathways
response to virus
defense response to virus
response to interferon−gamma
interferon−gamma−mediated signaling pathway
response to type I interferon
histone mRNA catabolic process
type I interferon signaling pathway
regulation of defense response to virus
regulation of type I interferon production
negative regulation of type I interferon production
JAK−STAT cascade
positive regulation of interferon−alpha production
process utilizing autophagic mechanism
autophagy
regulation of JAK−STAT cascade
response to interferon−alpha
MyD88−dependent toll−like receptor signaling pathway
−log10 q value

Supplemental Figure 2. Bioinformatic characterization of RNA sequencing conducted in human monocytes following *in vivo* NR supplementation.

(A-F) Volcano plots showing all the genes from multiple comparisons under different metabolic states or activation states. The statistically significant genes were highlighted in red circles. "0" or "1" depicts naïve or activated monocytes, respectively. i.e., Refed0P indicating refed, naïve monocytes from placebo group. (G–L) PCA plots of DE genes from NR vs placebo or fasted vs refed comparison showing that NR supplementation or nutrient load was the principal component driving gene expression changes, irrespective of monocyte activation status. Pathway enrichment analysis of the DE genes comparing NR group vs. placebo group from the naïve monocytes (M) and activated monocytes (N) under the refed state. The x axis represents negative log 10 transformed q values, and the bar plot color is scaled to the transformed q values.

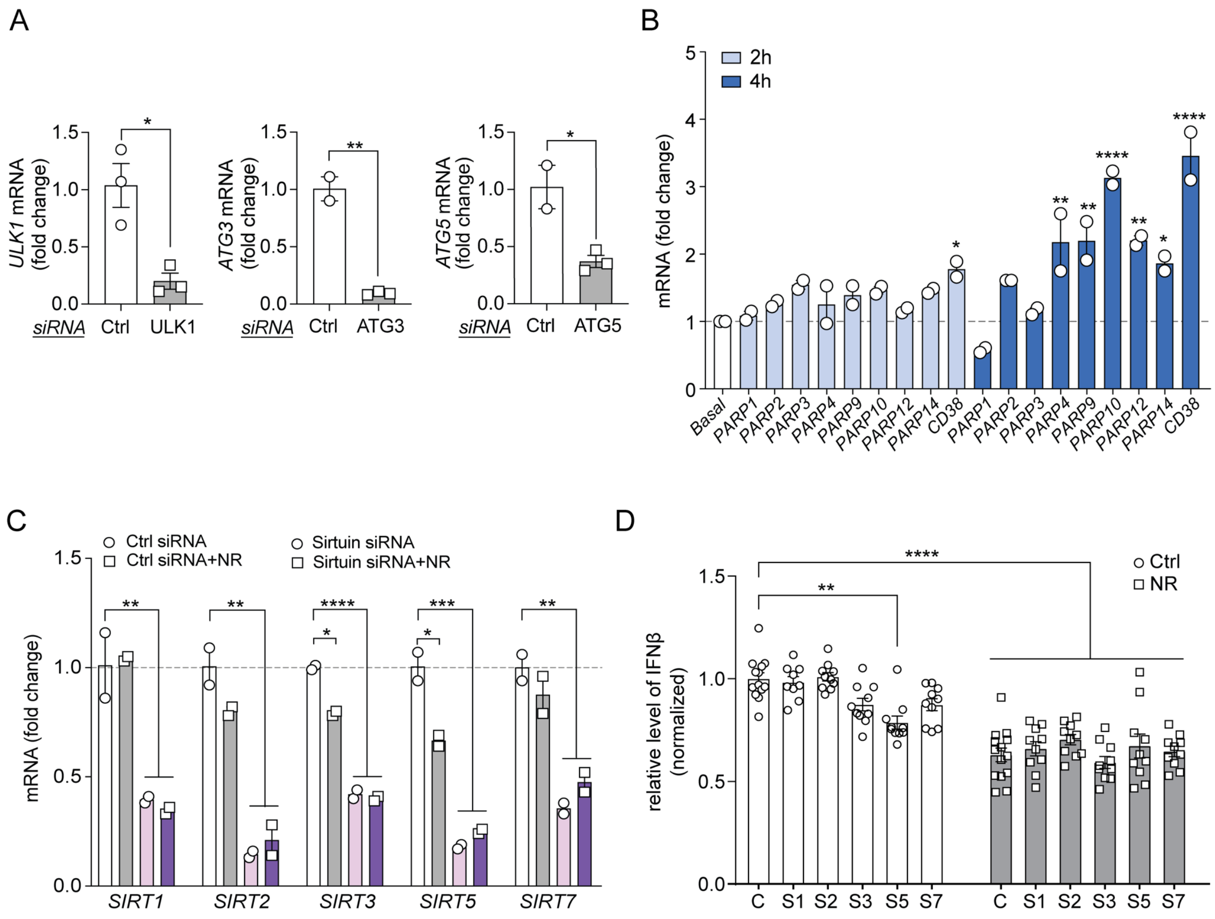

Supplemental Figure 3. LPS increases CD38/PARPs mRNA levels and Sirtuin depletion did not modulate NR-mediated attenuation of IFNβ release induced by LPS.

(A) Relative expression of ULK1, ATG3 and ATG5 was measured by quantitative RT-PCR showing autophagy related mRNAs were reduced by over 50% in human monocytes with siRNA nucleofection. (B) Relative expression of CD38 and PARPs mRNA in human monocytes upon LPS was measured by qRT-PCR. (B) Sirtuin mRNAs were reduced by over 50% in human monocytes with siRNA nucleofection. (C) IFNβ production induced by LPS (10ng/mL for 8hrs) was measured by ELISA in monocytes transfected with either control siRNA or sirtuin siRNA (n=10-13 replicates/treatment). Data were analyzed by one-way ANOVA followed by Dunnett's multiple comparisons test. All data were represented as mean ± SEM. **p*<0.05; ***p*<0.01; ****p*<0.001; *****p<0.0001;* ns, not significant.

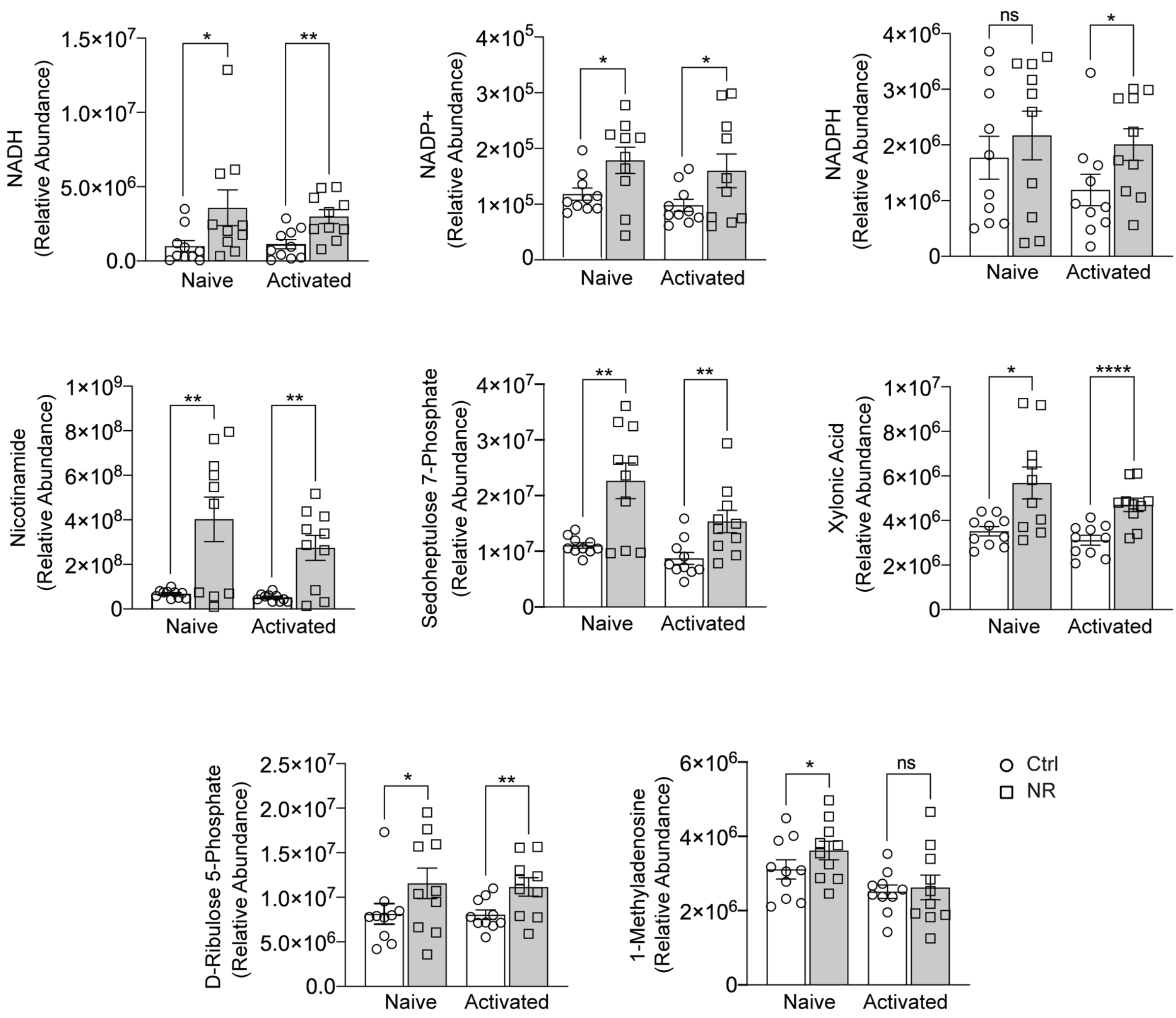

Supplemental Figure 4. Multiple metabolites involved in PPP, nicotinamide metabolism and Purine Metabolism are upregulated in NR supplemented groups.

Relative abundance of NADH, NADP+, NADPH, Niacinamide, Sedoheptulose 7-Phosphate, Xylonic acid, D-Ribulose 5-Phosphate, and 1-Methyladenosine in vehicle control and NR supplemented groups. Unpaired two-tailed Student's *t*-test. All data were represented as mean ± SEM. *$*p<0.05$; $**p<0.01$; $***p<0.001$; $****p<0.0001$;* ns, not significant.

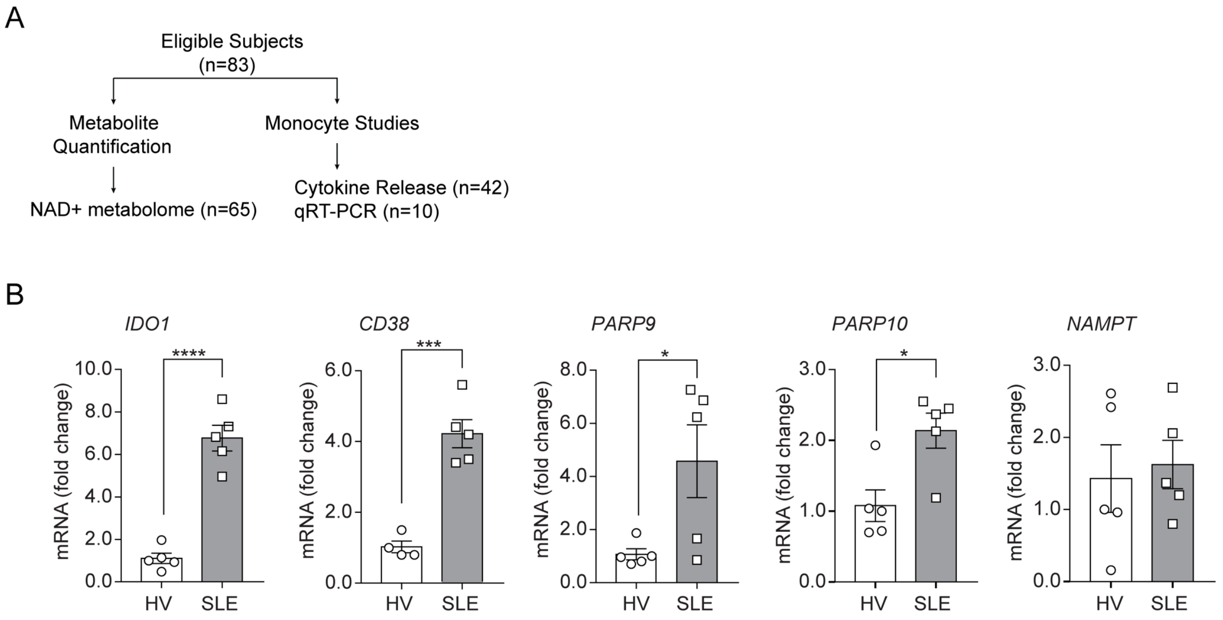

Supplemental Figure 5. Relative mRNA expression of enzymes involved in *de novo* NAD$^+$ synthesis and NAD$^+$ consumption is upregulated in monocytes from inactive Systemic Lupus Erythematosus patients (SLE).

(A) Flow diagram of human study comparing SLE patients with age/gender-matched healthy subjects. (B) Relative expression of enzymes involved in *de novo* NAD$^+$ synthesis (*IDO1*) and NAD$^+$ consumption (*CD38, PARP9,* and *PARP10*) was measured by quantitative RT-PCR in monocytes from healthy volunteers (HV) and inactive Systemic Lupus Erythematosus patients (SLE) (n=5 subjects/group). Unpaired two-tailed Student's *t*-test. Data were represented as mean ± SEM. **p<0.05; ***p<0.001; ****p<0.001.*

Supplemental Table 1. Research subjects' characteristics and laboratory results under baseline, fasted and refed states with Placebo or NR supplementation.

Characteristics	Metabolic State	Placebo	NR	*P* Value
**Subjects# (n)**	na	18	17	na
**Sex (M/F)**	na	6/12	8/9	na
**Age (y)**	na	23.67 ± 1.53	24.24 ± 2.10	0.38
**Race (n)**	na	W(11), B(4), A(3)	W(8), B(3), A(6)	na
**BMI (kg/m^2)**	na	22.98 ± 2.43	23.53 ± 2.64	0.54
**Glucose (mg/dL)**	Baseline	87.61±9.01	90.76±8.81	0.32
	Fasted	78.50 ± 9.85	87.12 ± 7.28	0.007**
	Refed	82.33 ± 13.21	85.18 ± 9.33	0.48
**Growth Hormones (ng/mL)**	Fasted	5.03 ± 4.77	3.40 ± 4.45	0.33
	Refed	1.85 ± 2.36	1.65 ± 2.44	0.82
**Insulin (mcU/mL)**	Fasted	4.38 ± 2.18	7.32 ± 3.36	0.005**
	Refed	15.87 ± 11.73	11.04 ± 5.91	0.15

Data are represented as mean ± standard deviation (SD). Abbreviations for race: W – White, B – Black, A – Asian. The *p* values for comparisons of two groups (placebo *vs.* NR under the same metabolic state were calculated using unpaired two-tailed Student's *t*-test. **p<0.01. na, not applicable. BMI, body mass index.

Supplemental Table 2. Complete Blood Count in baseline, fasted and refed state in human subjects with Placebo or NR supplementation.

Blood Cell Type	Metabolic State	Placebo ($x10^3/mL$) (n=18)	NR ($x10^3/mL$) (n=17)	Placebo (%) (n=18)	NR (%) (n=17)
**WBC**	Baseline	5.59±1.37	5.71±1.27	n.a.	n.a.
	Fasted	6.14±2.55	4.63±1.10 *	n.a.	n.a.
	Refed	5.21±1.17	5.00±1.06	n.a.	n.a.
**Lymphocytes**	Baseline	1.98±0.38	1.70±0.36 *	36.77±7.98	30.97±7.97 *
	Fasted	1.98±0.41	1.82±0.46	35.76±10.83	40.51±10.90
	Refed	2.02±0.41	1.70±0.31	39.49±6.60	34.94±7.65
**Monocytes**	Baseline	0.46±0.17	0.49±0.15	8.11±2.27	8.76±2.09
	Fasted	0.44±0.17	0.44±0.16	7.44±1.74	9.68±3.15 *
	Refed	0.43±0.12	0.41±0.11	8.46±2.08	8.50±2.84
**Neutrophils**	Baseline	2.94±1.12	3.32±1.10	48.77±13.51	56.89±9.23
	Fasted	3.53±2.29	2.18±0.89	53.51±12.04	45.92±11.44
	Refed	2.58±0.82	2.72±0.95	48.69±6.84	53.01±8.90
**Eosinophils**	Baseline	0.15±0.09	0.13±0.09	2.68±1.50	2.16±1.41
	Fasted	0.13±0.10	0.11±0.07	2.44±1.86	2.75±1.86
	Refed	0.13±0.06	0.12±0.08	2.52±1.15	2.39±1.52
**Basophils**	Baseline	0.04±0.01	0.05±0.02	0.68±0.21	0.81±0.32
	Fasted	0.03±0.01	0.04±0.02	0.59±0.20	0.82±0.44
	Refed	0.03±0.02	0.04±0.02	0.57±0.30	0.76±0.32

Data are represented as mean ± standard deviation (SD). The *p* values for comparisons of two groups (placebo *vs.* NR; total number of WBC or % of total WBC) under the same metabolic state were calculated using unpaired two-tailed Student's *t*-test. *p<0.05. WBC, white blood count.

Supplemental Table 7. Characteristics of Healthy Volunteers and Systemic Lupus Erythematosus patients enrolled in the study.

Characteristics	Healthy Volunteers (HV)	Systemic Lupus Erythematosus (SLE)
**Subjects# (n)**	23	60
**Sex (M or F)**	F	F(57)/M(3)
**Age (y)**	46.00 ± 10.06	47.92 ±11.08
**SLEDAI**	na	3.17± 4.04

Data are represented as mean ± standard deviation (SD). SLEDAI, Systemic Lupus Erythematosus Disease Activity Index. na, not applicable.

Supplemental Table 9. Customer Synthesized Primers for qRT-PCR.

OLIGONUCLEOTIDES	SOURCE	IDENTIFIER
JAK2 Primers: Fwd: 5' TCTGGGGAGTATGTTGCAGAA 3'; Rev: 5' AGACATGGTTGGGTGGATACC 3'	This manuscript	N/A
STAT1 Primers: Fwd: 5' ATCAGGCTCAGTCGGGGAATA 3'; Rev: 5' TGGTCTCGTGTTCTCTGTTCT 3'	This manuscript	N/A
STAT2 Primers: Fwd: 5' CCAGCTTTACTCGCACAGC 3'; Rev: 5' AGCCTTGGAATCATCACTCCC 3'	This manuscript	N/A
CXCL10 Primers: Fwd: 5' GTGGCATTCAAGGAGTACCTC 3'; Rev: 5' TGATGGCCTTCGATTCTGGATT 3'	This manuscript	N/A
ISG15 Primers: Fwd: 5' CGCAGATCACCCAGAAGATCG 3'; Rev: 5' TTCGTCGCATTTGTCCACCA 3'	This manuscript	N/A
IFITM1 Primers: Fwd: 5' CCAAGGTCCACCGTGATTAAC 3'; Rev: 5' ACCAGTTCAAGAAGAGGGTGTT 3'	This manuscript	N/A
APOBEC3G Primers: Fwd: 5'GCATCGTGACCAGGAGTATGA 3'; Rev: 5' GTCAGGGTAACCTTCGGGT 3'	This manuscript	N/A
EIF2AK2 Primers: Fwd: 5' GCCGCTAAACTTGCATATCTTCA 3'; Rev: 5' TCACACGTAGTAGCAAAAGAACC 3'	This manuscript	N/A
OAS1 Primers: Fwd: 5' TGTCCAAGGTGGTAAAGGGTG 3'; Rev: 5' CCGGCGATTTAACTGATCCTG 3'	This manuscript	N/A
UBE2L6 Primers: Fwd: 5' TGGACGAGAACGGACAGATTT3'; Rev: 5' GGCTCCCTGATATTCGGTCTATT 3'	This manuscript	N/A
MX1 Primers: Fwd: 5' GTTTCCGAAGTGGACATCGCA 3'; Rev: 5' CTGCACAGGTTGTTCTCAGC 3'	This manuscript	N/A
SOCS1 Primers: Fwd: 5' CACGCACTTCCGCACATTC 3'; Rev: 5' TAAGGGCGAAAAAGCAGTTCC 3'	This manuscript	N/A
CD38 Primers: Fwd: 5' AGACTGCCAAAGTGTATGGGA 3'; Rev: 5' GCAAGGTACGGTCTGAGTTCC 3'	This manuscript	N/A
IDO1 Primers: Fwd: 5' GCCAGCTTCGAGAAAGAGTTG 3'; Rev: 5'ATCCCAGAACTAGACGTGCAA 3'	This manuscript	N/A
NAMPT Primers: Fwd: 5' AATGTTCTCTTCACGGTGGAA AA 3'; Rev: 5' ACTGTGATTGGATACCAGGACT3'	This manuscript	N/A
PARP1 Primers: Fwd: 5' CGGAGTCTTCGGATAAGCTCT 3'; Rev: 5' TTTCCATCAAACATGGGCGAC 3'	This manuscript	N/A
PARP2 Primers: Fwd: 5' GGCACAAATCAAGGCAGGTTA3'; Rev: 5' AAGTCATGCGGAATCCTGGTG 3'	This manuscript	N/A
PARP3 Primers: Fwd: 5' GCCCTGGGTACAGACTGAG 3'; Rev: 5' CGCTTCTCTGCGGGTATGG 3'	This manuscript	N/A
PARP4 Primers: Fwd: 5' AGGTTTTGCAGAATCATCACAGT 3'; Rev: 5' CCTCACATTACCAAGTTTGCTCA 3'	This manuscript	N/A
PARP9 Primers: Fwd: 5' TCTGATGGGATTCAACGTGGA 3'; Rev: 5' TTCCTGGGCTGATAATTTCTGTG 3'	This manuscript	N/A
PARP10 Primers: Fwd: 5' CAGCTCTACCATGAGGACCTT 3'; Rev: 5' CGAAAGCCAGTCATATCCGGT 3'	This manuscript	N/A
PARP12 Primers: Fwd: 5' GCCATGACTTACGGTGCTACC 3'; Rev: 5' CCAAACTCATCACTCCAGTACCA 3'	This manuscript	N/A

Continued		
**OLIGONUCLEOTIDES**	**SOURCE**	**IDENTIFIER**
PARP14 Primers: Fwd: 5’ TGTTAGTGGAGAACATAAGTGGC 3’; Rev: 5’ TGAATGGTGCTTGGTACAATCAT 3’	This manuscript	N/A
ATG3 Primers: Fwd: 5’ ACATGGCAATGGGCTACAGG 3’; Rev: 5’ CTGTTTGCACCGCTTATAGCA 3’	This manuscript	N/A
ATP6V0C Primers: Fwd: 5’ TGAATGACGACATCAGCCTCT 3’; Rev: 5’ CGGCGAAGATGAGAATCAGGAT 3’	This manuscript	N/A
CD300A Primers: Fwd: 5’ CATCAACGTCAATGACACCTGC 3’; Rev: 5’ CACCCACTGCAAACAGGGTA 3’	This manuscript	N/A
LNPEP Primers: Fwd: 5’ AGTGCAACTGGTTACAGGCAG 3’; Rev: 5’ ACCACGATGACAAAAGCACAG 3’	This manuscript	N/A
PIKFYVE Primers: Fwd: 5’ GAACAGCAGCCTTTGAGTGGA 3’; Rev: 5’ GGTGTGGGTGACCTAACAGAC 3’	This manuscript	N/A
RAB3GAP1 Primers: Fwd: 5’ GCCTGGTAAGATGGTATGGGC 3’; Rev: 5’ GCACTTAGATTCGCTGAGAACA 3’	This manuscript	N/A
SCARB2 Primers: Fwd: 5’ AGATGGAGATTCTTTTCACCCAC 3’; Rev: 5’ CAGGAACTTTATACCGAAAGGCA 3’	This manuscript	N/A
SIDT2 Primers: Fwd: 5’ CAGTTCAGCTTCAATACCACAGC 3’; Rev: 5’ CCTTGACAATTACCGAGTCCAC 3’	This manuscript	N/A
TIGAR Primers: Fwd: 5’ ACTCAAGACTTCGGGAAAGGA 3’; Rev: 5’ CACGCATTTTCACCTGGTCC 3’	This manuscript	N/A
ULK3 Primers: Fwd: 5’ GAAGGACACTCGTGAAGTGGT3’; Rev: 5’ ACAATGTGGGGATGTCGAATG 3’	This manuscript	N/A
VPS33A Primers: Fwd: 5’ ATGGCGGCTCATCTGTCCTA 3’; Rev: 5’ CATCCCAAACTATTGCCTTGCT 3’	This manuscript	N/A

Supplemental Table 10. Wetern Blotting Antibodies Used in this study.

ANTIBODIES	SOURCE	IDENTIFIER
Rabbit monoclonal anti-IRF3[EPR2418Y]	Abcam	Cat# ab68481, RRID: AB_11155653
Rabbit monoclonal anti-IRF3 (phosphoS386) (EPR2346]	Abcam	Cat# ab76493, RRID: AB_1523836
Rabbit monoclonal anti-STAT1	Cell Signaling	Cat# 9172, RRID: AB_2198300
Rabbit monoclonal anti-STAT1(phosphoY701) (58D6)	Cell Signaling	Cat# 9167, RRID: AB_561284
Rabbit monoclonal anti-STAT2 (D9J7L)	Cell Signaling	Cat# 72604, RRID: AB_2799824
Rabbit monoclonal anti-STAT2(phosphoY690) (D3P2P)	Cell Signaling	Cat# 88410, RRID: AB_2800123
Rabbit monoclonal anti-TBK1/NAK (D1B4)	Cell Signaling	Cat# 3504, RRID: AB_2255663
Rabbit monoclonal anti-TBK1/NAK (phosphoS172) (D52C2)	Cell Signaling	Cat# 5483, RRID: AB_10693472
Mouse monoclonal anti-β-Actin (clone AC-15)	Sigma-Aldrich	Cat#: A2228, RRID: AB_476697
Rabbit polyclonal anti-LC3	Sigma-Aldrich	Cat#: L8918, RRID: AB_1079382
Rabbit monoclonal anti-LAMP1 (D2D11)	Cell Signaling	Cat#: 9091, RRID: AB_2687579
Rabbit monoclonal anti-SQSTM1/p62	Cell Signaling	Cat#: 5114, RRID: AB_10859911
Mouse monoclonal anti-Vinculin (VIN-11-5)	Sigma-Aldrich	Cat#: V4505, RRID: AB_477617
Rabbit monoclonal anti-Phospho-S6 Ribosomal Protein (Ser235/236)	Cell Signaling	Cat# 4858, RRID: AB_916156
